# Density functional theory study of doped coronene and circumcoronene as anode materials in lithium-ion batteries

**DOI:** 10.1038/s41598-024-66099-6

**Published:** 2024-07-02

**Authors:** Remya Geetha Sadasivan Nair, Arun Kumar Narayanan Nair, Shuyu Sun

**Affiliations:** Physical Science and Engineering Division (PSE), Computational Transport Phenomena Laboratory, King Abdullah University of Science and Technology (KAUST), 23955-6900 Thuwal, Saudi Arabia

**Keywords:** Theoretical chemistry, Density functional theory, Chemistry, Electrochemistry, Batteries

## Abstract

Density functional theory calculations are carried out to investigate the adsorption properties of Li^+^ and Li on twenty-four adsorbents obtained by replacement of C atoms of coronene (C_24_H_12_) and circumcoronene (C_54_H_18_) by Si/N/BN/AlN units. The molecular electrostatic potential (MESP) analysis show that such replacements lead to an increase of the electron-rich environments in the molecules. Li^+^ is relatively strongly adsorbed on all adsorbents. The adsorption energy of Li^+^ (E_ads-1_) on all adsorbents is in the range of − 42.47 (B_12_H_12_N_12_) to − 66.26 kcal/mol (m-C_22_H_12_BN). Our results indicate a stronger interaction between Li^+^ and the nanoflakes as the deepest MESP minimum of the nanoflakes becomes more negative. A stronger interaction between Li^+^ and the nanoflakes pushes more electron density toward Li^+^. Li is weakly adsorbed on all adsorbents when compared to Li^+^. The adsorption energy of Li (E_ads-2_) on all adsorbents is in the range of − 3.07 (B_27_H_18_N_27_) to − 47.79 kcal/mol (C_53_H_18_Si). Assuming the nanoflakes to be an anode for the lithium-ion batteries, the cell voltage (*V*_cell_) is predicted to be relatively high (> 1.54 V) for C_24_H_12_, C_12_H_12_Si_12_, B_12_H_12_N_12_, C_27_H_18_Si_27_, and B_27_H_18_N_27_. The E_ads-1_ data show only a small variation compared to E_ads-2_, and therefore, E_ads-2_ has a strong effect on the changes in V_cell_.

## Introduction

There is an increasing demand for rechargeable energy storage devices such as lithium ion batteries (LIBs) for their application in portable electronic devices and electric vehicles^1–3^. Since LIBs were first commercialized by Sony Corporation in the 1990s, they have occupied a dominant position in the portable electronics and automobile markets because of their high energy density and long service life^1–3^. Carbon materials such as graphene, carbon nanotube, carbon nanofiber, and polycyclic aromatic hydrocarbon (PAH) are widely used to construct anode materials in LIBs^4–9^. For example, graphene, a two-dimensional sheet of sp^2^-hybridized carbon atoms, has been proved to be a good electrode material for LIBs owing to its high surface area, high electrical conductivity, and superior mechanical flexibility. Doped carbonaceous materials were also used to construct anode materials in LIBs^10–14^.

PAHs are composed of two or more fused benzene rings and have many delocalized π electrons. Researchers have designed and synthesized various heteroatom-embedded PAHs to modify the π-electron properties^15–17^. Planar PAHs such as coronene (C_24_H_12_) and circumcoronene (C_54_H_18_) consist of seven and nineteen fused benzene rings, respectively. They can be considered as small portions of a graphene sheet with hydrogenated edges. There have been DFT studies on the adsorption properties of coronene, circumcoronene and their doped analogues^18–30^. The presence of silicon atoms in coronene favored its interaction with thiophene and similar compounds^18^. The hydrophobic ionic liquid adsorption on coronene and circumcoronene was stronger than that for the hydrophilic ionic liquid^19^. The free energy of adsorption of ionic liquids on BN-circumcoronene (B_27_H_18_N_27_) was negative, and thus, the adsorption occurred spontaneously^20^. The adsorption of organic molecules on Cu-doped coronene and circumcoronene was exothermic^21^. The relative positions of nitrogen and boron substitutions in the coronene gave different stabilities and different responses to the CO adsorption^22^. The interaction energies usually increased with the cluster size of the palladium atoms adsorbed on the coronene^23^. The doping of coronene with nitrogen atoms resulted in superior performance for CO_2_ and H_2_ adsorption^24^. The DFT studies on the adsorption of Li^+^ and Li on coronene, circumcoronene and BN-circumcoronene have recently attracted increasing interest. It was found that Li^+^/Li binds to the peripheral rings of coronene and circumcoronene^25–27^. Based on the cell voltage data, coronene was suggested as a good anode material for LIBs^27^. The adsorption energy of Li atom on B_27_H_18_N_27_ was found to be lower than that of circumcoronene^28^. However, the adsorption of Li^+^ and Li on adsorbents such as BN doped coronene is yet to be studied.

In this work, DFT calculations are performed to investigate the adsorption properties of Li^+^ and Li on twenty-four adsorbents obtained by replacement of C atoms of C_24_H_12_ and C_54_H_18_ by Si/N/BN/AlN units. The most-negative valued molecular electrostatic potential (MESP) point of molecules (V_min_) indicates the electron-rich regions such as π-region and lone-pair region^31,32^. A key observation is that the adsorption energies of Li^+^ on the nanoflakes are linearly correlated with the MESP *V*_min_ values of the nanoflakes. The V_cell_ is predicted to be relatively high (> 1.54 V) for C_24_H_12_, C_12_H_12_Si_12_, B_12_H_12_N_12_, C_27_H_18_Si_27_, and B_27_H_18_N_27_ The insights obtained from this work may be useful to design and explore better anode materials for LIBs.

## Computational details

All the DFT calculations were performed using the Gaussian 16 code^33^. In this work we consider a total of twenty-four adsorbents (C_24_H_12_ and its analogues C_23_H_12_Si, o-C_22_H_12_N_2_, m-C_22_H_12_N_2_, m-C_22_H_12_BN, p-C_22_H_12_BN, C_20_H_12_B_2_N_2_, C_18_H_12_B_3_N_3_, C_16_H_12_Si_8_, C_12_H_12_Si_12_, B_12_H_12_N_12_, B_11_H_12_N_12_Al; C_54_H_18_ and its analogues C_53_H_18_Si, o-C_52_H_18_N_2_, m-C_52_H_18_N_2_, m-C_52_H_18_BN, p-C_52_H_18_BN, C_50_H_18_B_2_N_2_, C_48_H_18_B_3_N_3_, C_36_H_18_Si_18_, C_27_H_18_Si_27_, B_27_H_18_N_27_, B_26_H_18_N_27_Al) and two adsorbates (Li^+^ and Li). The other adsorbents are designed by the replacements of C atoms in coronene and circumcoronene by Si/N/BN/AlN units. Here we also employed structural isomers of, for example, C_22_H_12_N_2_ (the two N atoms occupy adjacent positions in o-C_22_H_12_N_2_). Furthermore, C_16_H_12_Si_8_, C_12_H_12_Si_12_, and B_12_H_12_N_12_ can be considered as small portions of SiC_2_, SiC, and BN graphene-like sheets, respectively. The same applies to C_36_H_18_Si_18_, C_27_H_18_Si_27_, and B_27_H_18_N_27_. All structures were optimized at the M062X/6-31G(d,p) level^34^ and confirmed to be energy minima by frequency analysis. The MESP function V(r)^35–37^ is computed at the M062X/6-31G(d,p) level of theory using the below equation:

1 $$V\left(\textbf{r}\right)=\sum_{A=1}^{N}\frac{{Z}_{A}}{|{\textbf{r}-\textbf{R}}_{A}|}-\int \frac{\rho ({\textbf{r}}^{{{\prime}}})}{|{\textbf{r}-\textbf{r}}^{{{\prime}}}|}{\text{d}}^{3}{\textbf{r}}^{{{\prime}}}$$

where *Z*_*A*_ is the charge on nucleus A located at **R**_*A*_ and *ρ*(**r**) is the electronic charge density. The first term on the right-hand side of Eq. (1) stands for the nuclear contribution and the second term stands for the electronic contribution. *V*(r) is positive if the nuclear effects dominate, while it is negative if the electronic effects dominate.

**Figure 1:**
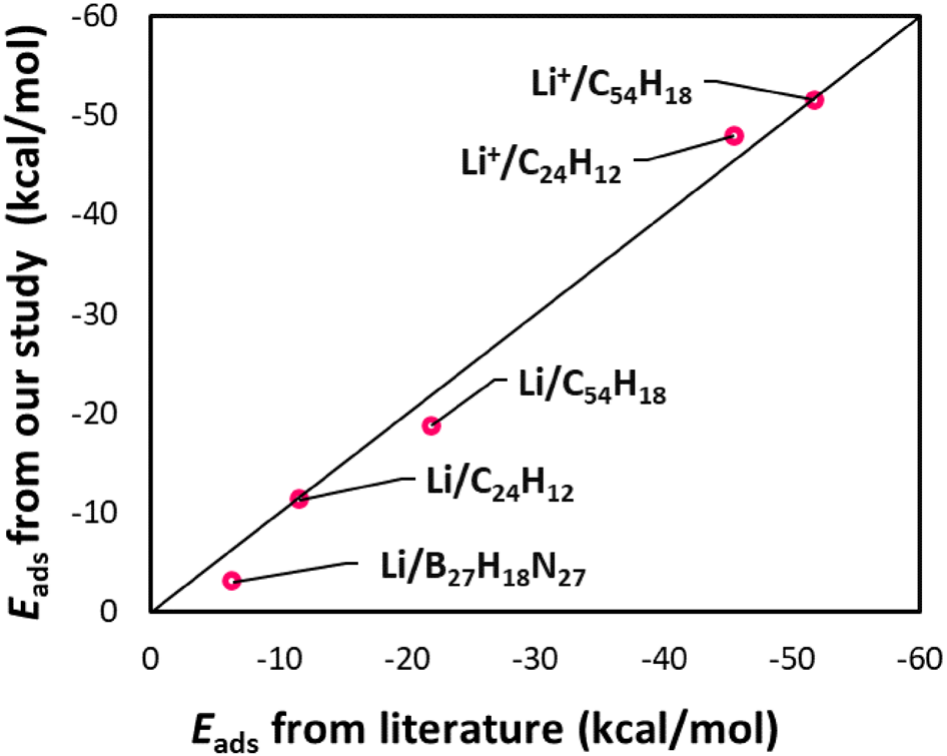
Comparison of our results for adsorption energies with literature values^26–28^.

The adsorption energy (*E*_ads_) is evaluated according to the formula:

2 $$E_{{{\text{ads}}}} = E_{{{\text{adsorbate }}/{\text{nanoflake}}}} {-} \, \left( {E_{{{\text{nanoflake}}}} + E_{{{\text{adsorbate}}}} } \right) \, + {\text{ E}}_{{{\text{BSSE}}}}$$

where *E*_adsorbate/nanoflake_, *E*_nanoflake_, and *E*_adsorbate_ represent the energy of adsorbed system, adsorbent (e.g., coronene), and adsorbate (Li^+^ or Li), respectively. E_BSSE_ is the basis set superposition error energy determined by the counterpoise approach^38^. The *E*_ads_ of Li^+^ and Li on the nanoflakes is represented as *E*_ads-1_ and *E*_ads-2_, respectively. Our calculated E_ads-1_ and E_ads-1_ values are consistent with previous results^26–28^ (Fig. 1).

The highest occupied molecular orbital (HOMO)-lowest unoccupied molecular orbital (LUMO) energy gap (E_g_) is calculated as follows:

3 $${\text{E}}_{{\text{g}}} = {\text{ E}}_{{{\text{LUMO}}}} - {\text{E}}_{{{\text{HOMO}}}}$$

where E_HOMO_ and E_LUMO_ represent the energy of HOMO and LUMO, respectively. The *E*_g_ of the pristine nanoflakes, Li^+^-adsorbed nanoflakes, and Li-adsorbed nanoflakes is represented as E_g-1_, E_g-2_, and E_g-3_, respectively.

The percentage change in the HOMO–LUMO energy gap is estimated as:

4 $$\Delta {\text{E}}_{{{\text{g}} - {1}}} = \, [({\text{E}}_{{{\text{g}} - {2}}} - {\text{E}}_{{{\text{g}} - {1}}} )/{\text{E}}_{{{\text{g}} - {1}}} ] \times {1}00$$

5 $$\Delta {\text{E}}_{{{\text{g}} - {2}}} = [({\text{E}}_{{{\text{g}} - {3}}} - {\text{E}}_{{{\text{g}} - {1}}} )/{\text{E}}_{{{\text{g}} - {1}}} ] \times {1}00$$

Assuming the nanoflake to be an anode for the LIBs, we can write the reaction in the anode and cathode as follows:

6 $${\text{Anode}}{:}\;\;{\text{ Li}}/{\text{nanoflake}} \leftrightarrow {\text{ Li}}^{ + } /{\text{nanoflake }} + {\text{e}}^{ - }$$

7 $${\text{Cathode}}{:}\;\;{\text{ Li}}^{ + } + {\text{ e}}^{ - } \leftrightarrow {\text{ Li}}$$

Thus, the total cell reaction can be written as follows:

8 $${\text{Li}}/{\text{nanoflake}} + {\text{ Li}}^{ + } \leftrightarrow {\text{ Li}}^{ + } /{\text{nanoflake }} + {\text{ Li }} + \Delta {\text{G}}_{{{\text{cell}} }}$$

Here ∆G_cell_ is the Gibbs free energy change for the total cell reaction, which can be expressed as

9 $$\Delta {\text{G}}_{{{\text{cell}}}} = \, \Delta {\text{E}}_{{{\text{cell}}}} + {\text{P}}\Delta {\text{V}}{-}{\text{T}}\Delta {\text{S}}$$

where

10 $$\Delta {\text{E}}_{{{\text{cell}}}} = {\text{ E}}_{{\text{ads - 1}}} - {\text{ E}}_{{\text{ads - 2}}}$$

The cell voltage can be calculated using the Nernst equation:

11 $${\text{V}}_{{{\text{cell}}}} = \, - \Delta {\text{G}}_{{{\text{cell}}}} /{\text{zF}}$$

where F and z are the Faraday constant (96,485.3 C/mol) and the charge of Li^+^, respectively. It may be assumed that ΔG_cell_ ≈ ΔE_cell_, since the contribution of entropy and volume effects to V_cell_ is expected to be very small^39^.

## Results and discussion

### Coronene and its analogues as anode materials in lithium-ion batteries

#### Structural properties of coronene and its analogues

**Figure 2:**
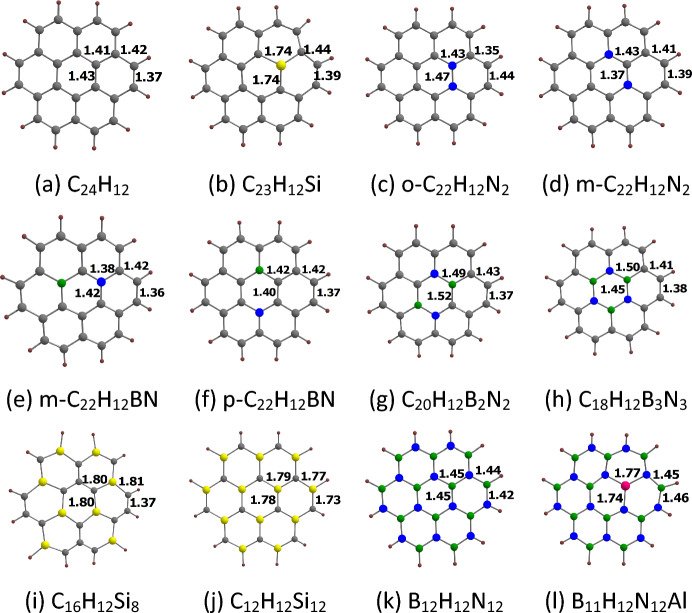
Optimized structures of (**a**) C_24_H_12_, (**b**) C_23_H_12_Si, (**c**) o-C_22_H_12_N_2_, (**d**) m-C_22_H_12_N_2_, (**e**) m-C_22_H_12_BN, (**f**) p-C_22_H_12_BN, (**g**) C_20_H_12_B_2_N_2_, (**h**) C_18_H_12_B_3_N_3_, (**i**) C_16_H_12_Si_8_, (**j**) C_12_H_12_Si_12_, (**k**) B_12_H_12_N_12_, (**l**) B_11_H_12_N_12_Al. The bond distances are given in Å. Color code: gray-C, green-B, blue-N, purple-Al, yellow-Si.

The optimized structures of coronene and its analogues are given in Fig. 2, indicating all the important bond length values. The XY (X = C, Si, N, B, Al; Y = C, Si, N, B, Al) bond lengths of coronene and its analogues are in the range of about 1.35 to 1.81 Å. In comparison to the CC bond length in benzene (1.395 Å)^40^, we see that the CC bond lengths in coronene are in the range of about 1.37 to 1.43 Å. The six outermost CC bonds in coronene are noticeably shorter (1.37 Å) than the other ones. Their bond lengths are close to the CC bond length in ethylene (1.330 Å)^41^, indicating the significant double bond character of these bonds. Here the CSi (> 1.73 Å) and NAl (> 1.74 Å) bond lengths are found to be significantly longer than all the CC bond lengths in coronene.

The E_HOMO,_ E_LUMO_, and E_g-1_ values of coronene and its analogues are given in Table 1. The molecular orbital diagrams for some representative nanoflakes are shown in Fig. S1. Our results show that the E_HOMO,_ E_LUMO_, and E_g-1_ values of coronene and its analogues are in the ranges of − 8.27 (B_12_H_12_N_12_) to − 3.82 eV (m-C_22_H_12_N_2_), − 1.43 (p-C_22_H_12_BN) to 1.24 eV (B_12_H_12_N_12_), and 2.95 (m-C_22_H_12_N_2_) to 9.51 eV (B_12_H_12_N_12_), respectively. The E_HOMO_, E_LUMO_, and E_g-1_ values of coronene are − 6.62, − 0.72, and 5.90 eV, respectively. In general, the E_HOMO_ (E_LUMO_) of the doped coronene is higher (lower) than that of the pristine coronene. Also, the E_g-1_ of the doped coronene is generally lower than that of the pristine coronene. The opposite trends are observed for the B_12_H_12_N_12_ and B_11_H_12_N_12_Al. Typically, a smaller HOMO–LUMO energy gap indicates a better electronic conductivity. These E_HOMO,_ E_LUMO_, and E_g-1_ values reported here are consistent with previous theoretical results^29^.

**Table 1:** *V*_min,_ E_HOMO,_ E_LUMO_ and E_g-1_ for doped coronenes^a^.

Nanoflake	*V*_min_	E_HOMO_	E_LUMO_	E_g-1_
C_24_H_12_	− 16.06	− 6.62	− 0.72	5.90
C_23_H_12_Si	− 19.70	− 6.34	− 1.04	5.30
o-C_22_H_12_N_2_	− 24.22	− 4.61	− 0.74	3.87
m-C_22_H_12_N_2_	− 21.34	− 3.82	− 0.88	2.95
m-C_22_H_12_BN	− 31.38	− 5.89	− 1.35	4.53
p-C_22_H_12_BN	− 30.43	− 5.82	− 1.43	4.39
C_20_H_12_B_2_N_2_	− 22.15	− 6.24	− 1.06	5.18
C_18_H_12_B_3_N_3_	− 15.44	− 6.08	− 1.16	4.93
C_16_H_12_Si_8_	− 26.73	− 5.87	− 1.41	4.46
C_12_H_12_Si_12_	− 24.72	− 6.46	− 1.01	5.45
B_12_H_12_N_12_	− 16.25	− 8.27	1.24	9.51
B_11_H_12_N_12_Al	− 21.52	− 8.25	0.28	8.53

^a^The values are given in eV and *V*_min_ in kcal/mol.

#### MESP of coronene and its analogues

**Figure 3:**
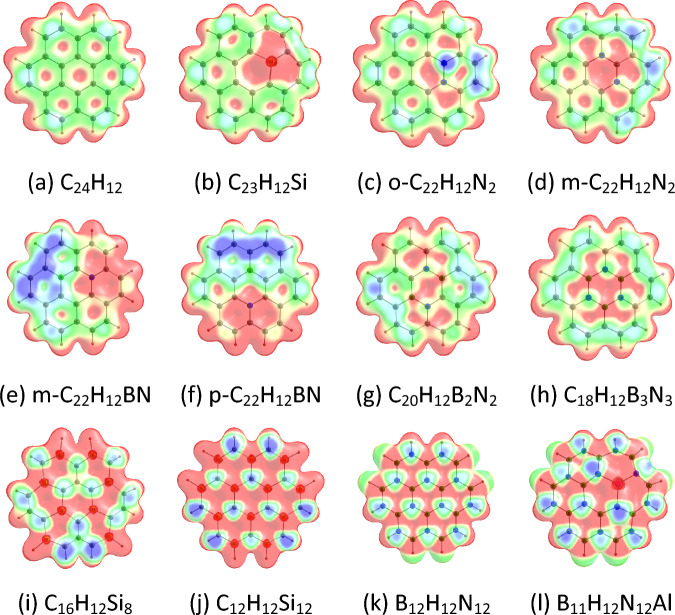
MESP mapped on 0.01 a.u. electron density isosurface of (**a**) C_24_H_12_, (**b**) C_23_H_12_Si, (**c**) o-C_22_H_12_N_2_, (**d**) m-C_22_H_12_N_2_, (**e**) m-C_22_H_12_BN, (**f**) p-C_22_H_12_BN, (**g**) C_20_H_12_B_2_N_2_, (**h**) C_18_H_12_B_3_N_3_, (**i**) C_16_H_12_Si_8_, (**j**) C_12_H_12_Si_12_, (**k**) B_12_H_12_N_12_, (**l**) B_11_H_12_N_12_Al. The colour coding from blue to red indicates MESP values in the range − 0.03 to 0.03 a.u. The colours at the blue end of the spectrum indicate the electron-rich regions, while those toward the red indicate the electron-deficient regions.

The MESP features of coronene and its analogues are given in Fig. 3. The MESP analysis suggest that electron-rich regions (e.g., green regions) are present on coronene due to cyclic π-electron delocalization. We see that the replacements of C atoms of coronene by Si/N/BN/AlN units lead to an overall increase of the electron-rich environments (e.g., blue regions) in the molecules. The MESP *V*_min_ values of coronene and its analogues (represented as *V*_min_) are provided in Table 1. The locations of *V*_min_ in the MESP plots of coronene and its analogues are provided in Fig. S2, electronic Supporting Information (ESI). The MESP *V*_min_ points are located near the peripheral rings of coronene. This shows that the peripheral rings of coronene are electron richer than its central ring. This is because the peripheral rings of coronene contain C–H bonds also and the sp^2^ carbon is more electronegative than H. For other adsorbents also, the MESP *V*_min_ points are located near the peripheral rings. We find that the MESP *V*_min_ values of coronene and its analogues are in the range of − 15.44 (C_18_H_12_B_3_N_3_) to − 31.38 kcal/mol (m-C_22_H_12_BN). The MESP *V*_min_ value of coronene is − 16.06 kcal/mol. The MESP *V*_min_ of the doped coronene is generally higher than that of the pristine coronene. However, the MESP *V*_min_ of C_18_H_12_B_3_N_3_ is slightly lower than that of coronene. For the N-doped coronenes, the MESP *V*_min_ value of o-C_22_H_12_N_2_ (− 24.22 kcal/mol) is higher than that of m-C_22_H_12_N_2_ (− 21.34 kcal/mol). The MESP *V*_min_ values of Si-doped coronenes increase in the order C_23_H_12_Si < C_12_H_12_Si_12_ < C_16_H_12_Si_8_. Furthermore, the MESP *V*_min_ values of BN/AlN-doped coronenes increase in the order C_18_H_12_B_3_N_3_ < B_12_H_12_N_12_ < B_11_H_12_N_12_Al < C_20_H_12_B_2_N_2_ < p-C_22_H_12_BN < m-C_22_H_12_BN.

#### Li^+^ adsorption on the doped coronenes

**Figure 4:**
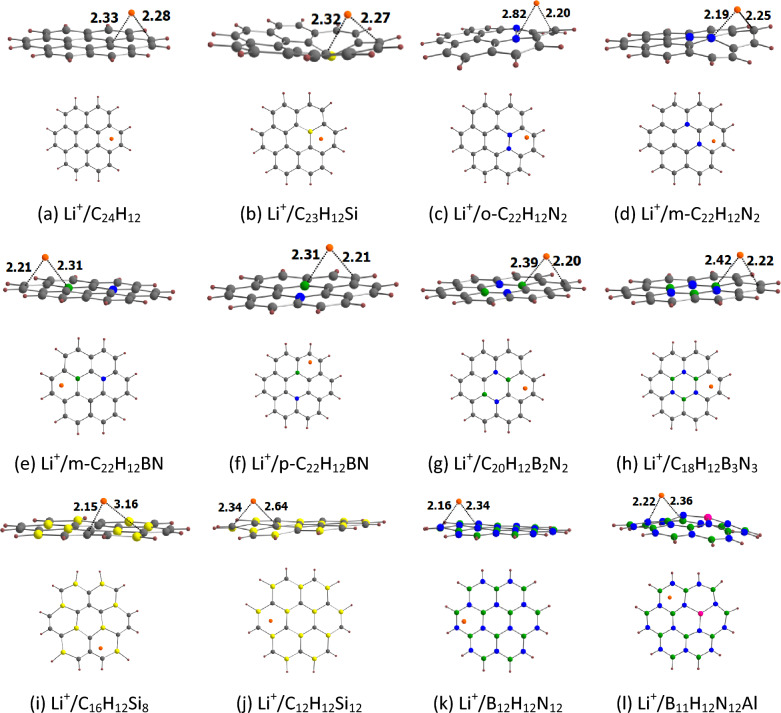
Optimized structures of Li^+^ adsorbed on (**a**) C_24_H_12_, (**b**) C_23_H_12_Si, (**c**) o-C_22_H_12_N_2_, (**d**) m-C_22_H_12_N_2_, (**e**) m-C_22_H_12_BN, (**f**) p-C_22_H_12_BN, (**g**) C_20_H_12_B_2_N_2_, (**h**) C_18_H_12_B_3_N_3_, (**i**) C_16_H_12_Si_8_, (**j**) C_12_H_12_Si_12_, (**k**) B_12_H_12_N_12_, (**l**) B_11_H_12_N_12_Al. The bond distances are given in Å. The color code is the same as in Fig. 2. In addition, Li is denoted by orange color.

**Figure 5:**
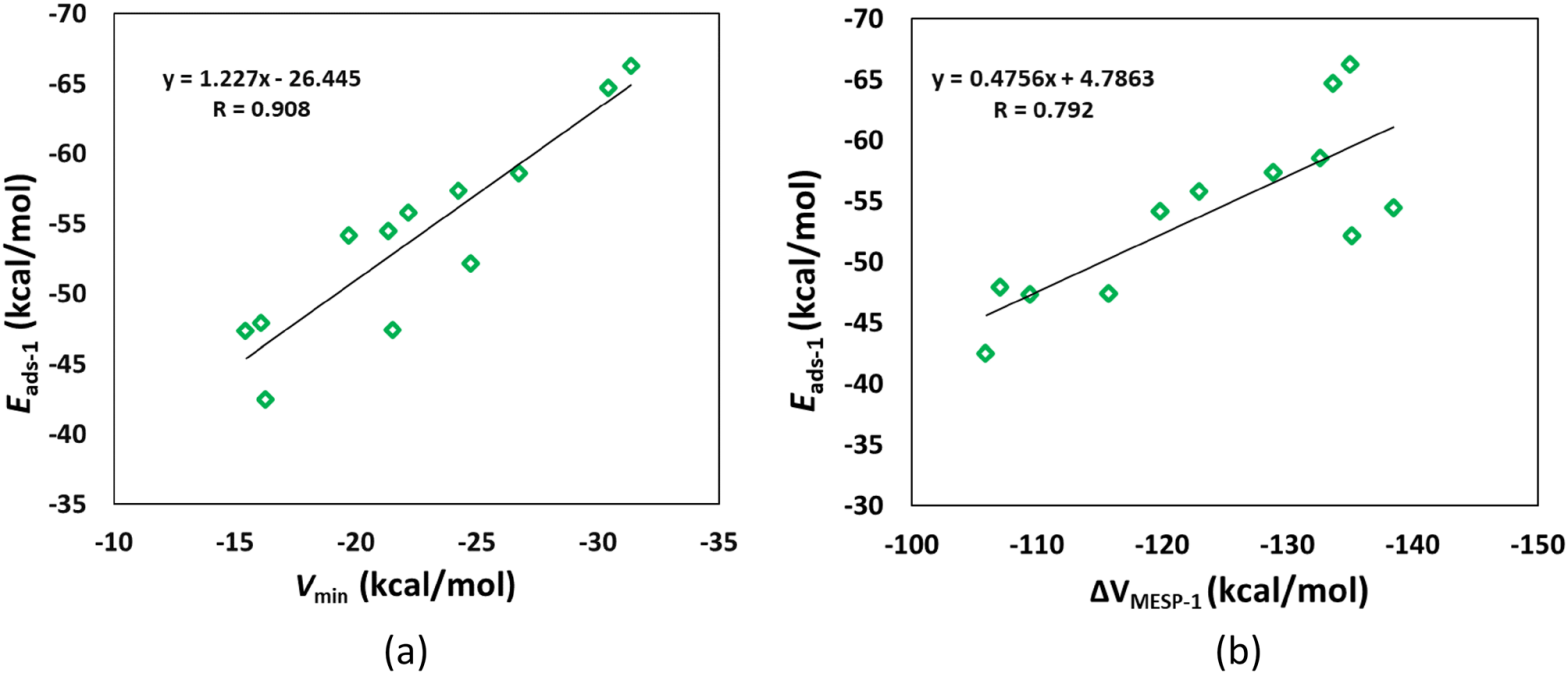
Correlation between (**a**) *V*_min_ and *E*_ads-1_ and (**b**) ∆*V*_MESP-1_ and *E*_ads-1_ for Li^+^-adsorbed coronenes.

The optimized geometries for the adsorption of Li^+^ on coronene and its analogues are provided in Fig. 4. Our results show that Li^+^ binds to the peripheral rings of coronene. This is in line with the fact the peripheral rings of coronene are electron richer than its central ring (see above). The adsorption process here involves the cation–π interaction^27,42^. This interaction is basically of electrostatic origin because a positively charged cation interacts with the negatively charged electron cloud of π-systems. For other adsorbents also, Li^+^ binds to the peripheral rings. It can be seen that Li^+^ is relatively strongly adsorbed on coronene and its analogues. For example, the adsorption distance of Li^+^ on these adsorbents is in the range of 2.15 (C_16_H_12_Si_8_) to 2.34 Å (C_12_H_12_Si_12_). This observation is further supported by the adsorption energy of Li^+^ on coronene and its analogues (Table 2). The E_ads-1_ values of Li^+^ on coronene and its analogues are in the range of − 42.47 (B_12_H_12_N_12_) to − 66.26 kcal/mol (m-C_22_H_12_BN). These E_ads-1_ values are negative, which implies that all of the adsorption processes were exothermic in nature. The more negative the E_ads-1_ value, the stronger the adsorption is. The E_ads-1_ value of Li^+^ on coronene is − 47.92 kcal/mol. For the N-doped coronenes, the E_ads-1_ value of o-C_22_H_12_N_2_ (− 57.33 kcal/mol) is higher than that of m-C_22_H_12_N_2_ (− 54.48 kcal/mol). The E_ads-1_ values of Si-doped coronenes increase in the order C_12_H_12_Si_12_ < C_23_H_12_Si < C_16_H_12_Si_8_. Furthermore, the E_ads-1_ values of BN/AlN-doped coronenes increase in the order B_12_H_12_N_12_ < C_18_H_12_B_3_N_3_ < B_11_H_12_N_12_Al < C_20_H_12_B_2_N_2_ < p-C_22_H_12_BN < m-C_22_H_12_BN. A key observation is that these adsorption energies are well correlated with the MESP *V*_min_ values of coronene and its analogues, with a correlation coefficient of 0.908 (Fig. 5a). These results indicate a stronger interaction between Li^+^ and the nanoflakes as the MESP *V*_min_ of the nanoflakes becomes more negative.

**Table 2:** E_ads-1_, Δ*V*_MESP-1_, E_HOMO_ E_LUMO,_ E_g-2_ and ∆E_g-1_ values for Li^+^ adsorbed coronenes^a^.

Nanoflake	E_ads-1_	Δ*V*_MESP-1_	E_HOMO_	E_LUMO_	E_g-2_	∆E_g-1_
C_24_H_12_	− 47.92	− 107.01	− 10.05	− 4.38	5.67	− 3.87
C_23_H_12_Si	− 54.14	− 119.84	− 10.08	− 5.06	5.02	− 5.1
o-C_22_H_12_N_2_	− 57.33	− 128.84	− 9.07	− 4.57	4.50	16.3
m-C_22_H_12_N_2_	− 54.48	− 138.49	− 8.18	− 4.46	3.73	26.3
m-C_22_H_12_BN	− 66.26	− 135.02	− 9.87	− 4.67	5.20	14.7
p-C_22_H_12_BN	− 64.72	− 133.65	− 9.79	− 4.77	5.02	14.4
C_20_H_12_B_2_N_2_	− 55.82	− 122.90	− 9.69	− 4.61	5.08	− 1.9
C_18_H_12_B_3_N_3_	− 47.32	− 109.44	− 9.51	− 4.71	4.80	− 2.5
C_16_H_12_Si_8_	− 58.57	− 132.63	− 9.11	− 4.61	4.50	1.0
C_12_H_12_Si_12_	− 52.14	− 135.13	− 9.22	− 4.20	5.02	− 7.9
B_12_H_12_N_12_	− 42.47	− 105.91	− 11.37	− 4.33	7.04	− 26.0
B_11_H_12_N_12_Al	− 47.40	− 115.74	− 11.25	− 4.25	7.00	− 18.0

^a^The values of E_ads-1_ and Δ*V*_MESP-1_ in kcal/mol ; E_HOMO_, E_LUMO_, and E_g-2_ in eV ; ∆E_g-1_ in %.

The electron donation from coronene and its analogues to Li^+^ could be checked by assessing the changes in the MESP at the nucleus of Li^+^ upon adsorption. Hence, the Δ*V*_MESP-1_ was obtained by taking the difference between the MESP at the nucleus of Li^+^ in the Li^+^-adsorbed nanoflake and the MESP at the free Li^+^ (− 5.36 au). Here the negative values of Δ*V*_MESP-1_ (Table 2) indicate the electron donation from coronene and its analogues to Li^+^. These Δ*V*_MESP-1_ values are in the range of − 105.91 (B_12_H_12_N_12_) to − 138.49 kcal/mol (m-C_22_H_12_N_2_). The Δ*V*_MESP-1_ value of coronene is − 107.01 kcal/mol. The Δ*V*_MESP-1_ of the doped coronenes is typically higher than that of the pristine coronene. However, the Δ*V*_MESP-1_ of B_12_H_12_N_12_ is lower than that of coronene. The adsorption energies of Li^+^ on coronene and its analogues are correlated with these Δ*V*_MESP-1_ values, with a correlation coefficient of 0.792 (Fig. 5b). These results indicate that a stronger interaction between Li^+^ and the nanoflakes pushes more electron density toward Li^+^.

The E_HOMO,_ E_LUMO_, and E_g-2_ values of Li^+^-adsorbed coronene and its analogues are given in Table 2. The E_HOMO,_ E_LUMO_, and E_g-2_ values of Li^+^-adsorbed coronene and its analogues are in the ranges of − 11.37 (B_12_H_12_N_12_) to − 8.18 eV (m-C_22_H_12_N_2_), − 5.06 (C_23_H_12_Si) to − 4.20 eV (C_12_H_12_Si_12_), and 3.73 (m-C_22_H_12_N_2_) to 7.04 eV (B_12_H_12_N_12_), respectively. Here, E_HOMO_ of the Li^+^-adsorbed nanoflakes is lower than that of the pristine nanoflakes for all cases (see Table 1). A similar result is obtained for E_LUMO_. The results here show that E_g-2_ is typically lower than E_g-1_. For example, the E_HOMO_, E_LUMO_, and E_g-2_ values of Li^+^ adsorbed coronene are − 10.05, − 4.38, and 5.67 eV, respectively. The decrease in the HOMO–LUMO energy gap for the adsorption of Li^+^ on coronene is 3.87% (see ΔE_g-1_ values in Table 2). A maximum decrease in the HOMO–LUMO energy gap of 26.0% was observed for the adsorption of Li^+^ on B_12_H_12_N_12_. However, E_g-2_ is found to be higher than E_g-1_ for the adsorption of Li^+^ on o-C_22_H_12_N_2_, m-C_22_H_12_N_2_, m-C_22_H_12_BN, p-C_22_H_12_BN, and C_16_H_12_Si_8_. A maximum increase in the HOMO–LUMO energy gap of 26.3% was observed for the adsorption of Li^+^ on m-C_22_H_12_N_2_.

#### Li adsorption on the doped coronenes

**Figure 6:**
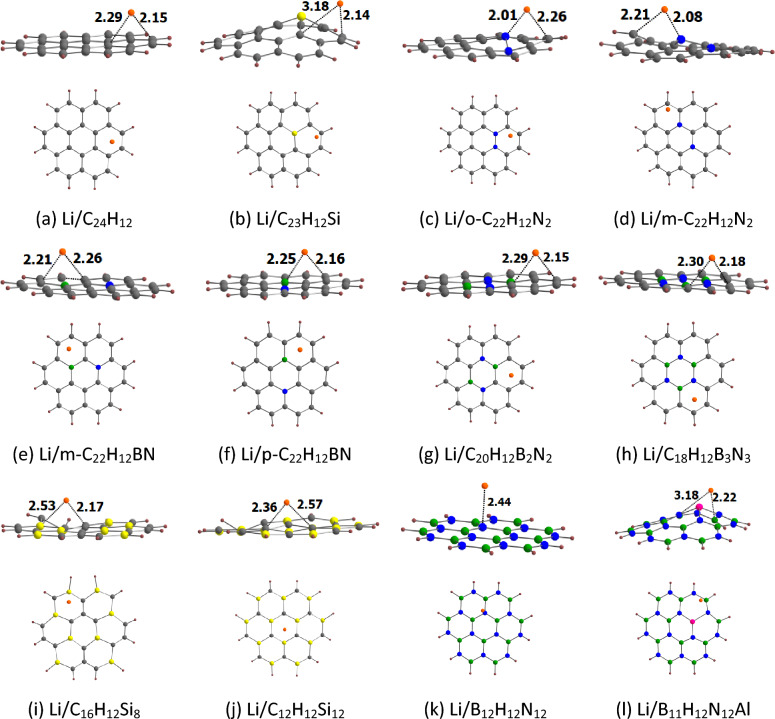
Optimized structures of Li adsorbed on (**a**) C_24_H_12_, (**b**) C_23_H_12_Si, (**c**) o-C_22_H_12_N_2_, (**d**) m-C_22_H_12_N_2_, (**e**) m-C_22_H_12_BN, (**f**) p-C_22_H_12_BN, (**g**) C_20_H_12_B_2_N_2_, (**h**) C_18_H_12_B_3_N_3_, (**i**) C_16_H_12_Si_8_, (**j**) C_12_H_12_Si_12_, (**k**) B_12_H_12_N_12_, (**l**) B_11_H_12_N_12_Al. The bond distances are given in Å. The color code is the same as in Fig. 4.

The optimized geometries for the adsorption of Li on coronene and its analogues are provided in Fig. 6. Our results show that Li mostly binds to the peripheral rings of coronene and its analogues, similar to the results obtained for Li^+^. The adsorption distance of Li on these adsorbents is in the range of 2.01 (o-C_22_H_12_N_2_) to 2.44 Å (B_12_H_12_N_12_). For these adsorbents, the adsorption distance of Li is generally smaller than that of Li^+^ (see Fig. 4). For example, the adsorption distance of Li and Li^+^ on coronene is 2.15 and 2.28 Å, respectively. However, Li is weakly adsorbed on coronene and its analogues when compared to Li^+^. This observation is supported by the adsorption energy of Li on coronene and its analogues (Table 3). The E_ads-2_ values of Li on coronene and its analogues are in the range of − 3.14 (B_12_H_12_N_12_) to − 37.63 kcal/mol (p-C_22_H_12_BN). The E_ads-2_ value of Li on coronene is − 11.32 kcal/mol. For the N-doped coronenes, the E_ads-2_ value of o-C_22_H_12_N_2_ (− 29.75 kcal/mol) is higher than that of m-C_22_H_12_N_2_ (− 23.69 kcal/mol). The E_ads-2_ values of Si-doped coronenes increase in the order C_12_H_12_Si_12_ < C_16_H_12_Si_8_ < C_23_H_12_Si. Furthermore, the E_ads-2_ values of BN/AlN-doped coronenes increase in the order B_12_H_12_N_12_ < C_18_H_12_B_3_N_3_ < B_11_H_12_N_12_Al < C_20_H_12_B_2_N_2_ < m-C_22_H_12_BN < p-C_22_H_12_BN. These adsorption energies are not well correlated with the MESP *V*_min_ values of coronene and its analogues (Fig. S3). The Δ*V*_MESP-2_ was obtained by taking the difference between the MESP at the nucleus of Li in the Li-adsorbed nanoflake and the MESP at the free Li (− 5.72 au). In general, the values of Δ*V*_MESP-2_ are positive for the adsorption of Li on coronene and its analogues (Table 3). The positive value of Δ*V*_MESP-2_ suggests the electron density transfer from Li to nanoflakes. However, the values of Δ*V*_MESP-2_ are negative for the adsorption of Li on o-C_22_H_12_N_2_, m-C_22_H_12_N_2_, C_16_H_12_Si_8_, and B_12_H_12_N_12_. Unlike E_ads-1_, E_ads-2_ does not show a correlation with Δ*V*_MESP-2_ (see Fig. S3).

**Table 3:** E_ads-2_, Δ*V*_MESP-2_, E_HOMO_ E_LUMO,_ E_g-3_ and ∆E_g-2_ values for Li adsorbed coronenes, along with ∆E_cell_ and V_cell_^a^.

Nanoflake	E_ads-2_	Δ*V*_MESP-2_	E_HOMO_	E_LUMO_	E_g-3_	∆E_g-2_	∆E_cell_	V_cell_
C_24_H_12_	− 11.32	17.58	− 3.30	− 0.94	2.37	− 59.9	− 36.60	1.59
C_23_H_12_Si	− 33.10	9.32	− 4.87	− 0.61	4.25	− 19.7	− 21.04	0.91
o-C_22_H_12_N_2_	− 29.75	− 1.23	− 3.91	− 0.72	3.19	− 17.6	− 27.59	1.20
m-C_22_H_12_N_2_	− 23.69	− 3.03	− 3.74	− 0.61	3.13	6.3	− 30.79	1.33
m-C_22_H_12_BN	− 37.20	6.86	− 3.87	− 0.78	3.08	− 32.0	− 29.06	1.26
p-C_22_H_12_BN	− 37.63	8.03	− 3.72	− 0.83	2.89	− 34.1	− 27.09	1.17
C_20_H_12_B_2_N_2_	− 24.84	9.40	− 3.56	− 0.89	2.67	− 48.3	− 30.98	1.34
C_18_H_12_B_3_N_3_	− 19.43	21.81	− 3.70	− 1.07	2.63	− 46.6	− 27.90	1.21
C_16_H_12_Si_8_	− 32.66	− 3.79	− 5.03	− 1.49	3.54	− 20.7	− 25.92	1.12
C_12_H_12_Si_12_	− 10.51	17.14	− 4.18	− 1.21	2.97	− 45.6	− 41.62	1.80
B_12_H_12_N_12_	− 3.14	− 21.13	− 3.24	0.56	3.81	− 60.0	− 39.33	1.71
B_11_H_12_N_12_Al	− 22.73	9.26	− 5.15	− 0.46	4.69	− 45.0	− 24.68	1.07

^a^The values of E_ads-2_, Δ*V*_MESP-2_, and ∆E_cell_ in kcal/mol ; E_HOMO_, E_LUMO_, and E_g-3_ in eV ; ∆E_g-2_ in % ; V_cell_ in V.

The E_HOMO,_ E_LUMO_, and E_g-3_ values of Li-adsorbed coronene and its analogues are given in Table 3. The E_HOMO,_ E_LUMO_, and E_g-3_ values of Li-adsorbed coronene and its analogues are in the ranges of − 5.15 (B_11_H_12_N_12_Al) to − 3.24 eV (B_12_H_12_N_12_), − 0.94 (C_24_H_12_) to 0.56 eV (B_12_H_12_N_12_), and 2.37 (C_24_H_12_) to 4.69 eV (B_11_H_12_N_12_Al), respectively. For all cases, E_HOMO_ of the Li-adsorbed nanoflakes is higher than that of the pristine nanoflakes (see Table 1). For example, the E_HOMO_ value of Li-adsorbed coronene is − 3.30 eV. The E_LUMO_ of coronene and its analogues is not much affected by the presence of Li. The results here show that E_g-3_ is usually lower than E_g-1_. The decrease in the HOMO–LUMO energy gap for the adsorption of Li on coronene is 59.9% (see ΔE_g-2_ values in Table 3). A maximum decrease in the HOMO–LUMO energy gap of 60.0% was observed for the adsorption of Li on B_12_H_12_N_12_. However, E_g-3_ is found to be higher than E_g-1_ for the adsorption of Li on m-C_22_H_12_N_2_ (ΔE_g-2_ of 6.3%).

#### Cell voltage of the doped coronenes

The ΔE_cell_ and V_cell_ of LIBs based on coronene and its analogues were calculated using eqs. (10 and 11), respectively, and their values are given in Table 3. Here the ∆E_cell_ and V_cell_ values are in the ranges of − 41.62 (C_12_H_12_Si_12_) to − 21.04 kcal/mol (C_23_H_12_Si) and 0.91 (C_23_H_12_Si) to 1.80 V (C_12_H_12_Si_12_), respectively. The more negative the ∆E_cell_ value, the higher the V_cell_ value of the nanoflakes. Therefore, the nanoflakes interacting strongly with Li^+^ and weakly with Li may serve as good candidates for the anode materials in LiBs. For example, the highest V_cell_ value of 1.80 V is observed for C_12_H_12_Si_12_ due to the high E_ads-1_ of − 52.14 kcal/mol (see Table 2) and low E_ads-2_ of − 10.51 kcal/mol (see Table 3). The lowest V_cell_ value of 0.91 V observed for C_23_H_12_Si can be attributed to a higher contribution from E_ads-2_ (E_ads-1_ of − 54.14 kcal/mol and E_ads-2_ of − 33.10 kcal/mol). The ∆E_cell_ and V_cell_ values of coronene are − 36.60 kcal/mol and 1.59 V, respectively. For the N-doped coronenes, the V_cell_ value of o-C_22_H_12_N_2_ (1.20 V) is lower than that of m-C_22_H_12_N_2_ (1.33 V). The V_cell_ values of Si-doped coronenes increase in the order C_23_H_12_Si < C_16_H_12_Si_8_ < C_12_H_12_Si_12_. Furthermore, the V_cell_ values of BN/AlN-doped coronenes increase in the order B_11_H_12_N_12_Al < p-C_22_H_12_BN < C_18_H_12_B_3_N_3_ < m-C_22_H_12_BN < C_20_H_12_B_2_N_2_ < B_12_H_12_N_12_. It can be observed that the E_ads-1_ data exhibit only a small variation compared to E_ads-2_, and therefore E_ads-2_ has a relatively strong influence on the variation of V_cell_. The linear correlation of E_ads-2_ with V_cell_ (R = 0.805) also supports the strong influence of E_ads-2_ on V_cell_ (Fig. S4).

### Circumcoronene and its analogues as anode materials in lithium-ion batteries

#### Structural properties of circumcoronene and its analogues

**Figure 7:**
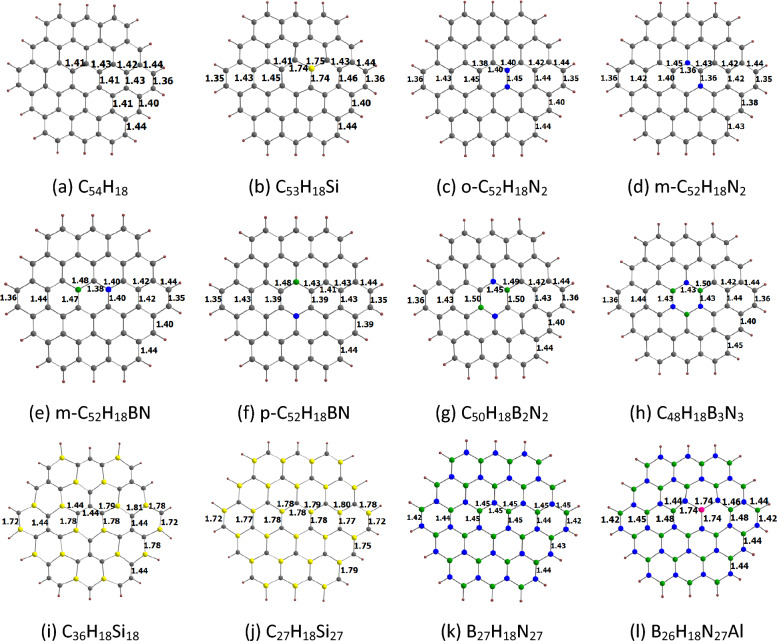
Optimized structures of (**a**) C_54_H_18_, (**b**) C_53_H_18_Si, (**c**) o-C_52_H_18_N_2_, (**d**) m-C_52_H_18_N_2_, (**e**) m-C_52_H_18_BN, (**f**) p-C_52_H_18_BN, (**g**) C_50_H_18_B_2_N_2_, (**h**) C_48_H_18_B_3_N_3_, (**i**) C_36_H_18_Si_18_, (**j**) C_27_H_18_Si_27_, (**k**) B_27_H_18_N_27_, (**l**) B_26_H_18_N_27_Al. The bond distances are given in Å. The color code is the same as in Fig. 2.

The optimized structures of circumcoronene and its analogues are given in Fig. 7, indicating all the important bond length values. As in the case of coronene and its analogues, the XY (X = C, Si, N, B, Al; Y = C, Si, N, B, Al) bond lengths of circumcoronene and its analogues are in the range of about 1.35 to 1.81 Å. We see that the CC bond lengths in circumcoronene are in the range of about 1.36 to 1.44 Å, and the six outermost CC bonds are noticeably shorter (1.36 Å) than the other ones. Here, all the CSi (> 1.72 Å) and NAl (≈1.74 Å) bond lengths are found to be significantly longer than all the CC bond lengths.

The E_HOMO,_ E_LUMO_, and E_g-1_ values of circumcoronene and its analogues are given in Table 4. Our results show that the E_HOMO,_ E_LUMO_, and E_g-1_ values of circumcoronene and its analogues are in the ranges of − 8.05 (B_27_H_18_N_27_) to − 4.03 eV (m-C_52_H_18_N_2_), − 2.00 (C_50_H_18_B_2_N_2_) to 1.08 eV (B_27_H_18_N_27_), and 2.14 (m-C_52_H_18_N_2_) to 9.12 eV (B_27_H_18_N_27_), respectively. The E_HOMO_, E_LUMO_, and E_g-1_ values of circumcoronene are − 5.95, − 1.60, and 4.35 eV, respectively. In general, the E_HOMO_ (E_LUMO_) of the doped circumcoronene is higher (lower) than that of the pristine circumcoronene. Also, the E_g-1_ of the doped circumcoronene is typically lower than that of the pristine circumcoronene. The opposite trends are observed for p-C_52_H_18_BN, C_48_H_18_B_3_N_3_, C_27_H_18_Si_27_, B_12_H_12_N_12_, and B_11_H_12_N_12_Al. These E_HOMO,_ E_LUMO_, and E_g-1_ values reported here are consistent with previous theoretical results^30^.

**Table 4:** *V*_min,_ E_HOMO,_ E_LUMO_ and E_g-1_ for doped circumcoronenes^a^.

Nanoflake	*V*_min_	E_HOMO_	E_LUMO_	E_g-1_
C_54_H_18_	− 14.56	− 5.95	− 1.60	4.35
C_53_H_18_Si	− 16.57	− 5.77	− 1.81	3.96
o-C_52_H_18_N_2_	− 17.19	− 4.65	− 1.60	3.04
m-C_52_H_18_N_2_	− 24.03	− 4.03	− 1.90	2.14
m-C_52_H_18_BN	− 22.34	− 5.71	− 1.79	3.92
p-C_52_H_18_BN	− 21.21	− 5.99	− 1.54	4.45
C_50_H_18_B_2_N_2_	− 16.57	− 5.42	− 2.00	3.41
C_48_H_18_B_3_N_3_	− 14.93	− 6.15	− 1.34	4.80
C_36_H_18_Si_18_	− 28.24	− 5.61	− 2.00	3.61
C_27_H_18_Si_27_	− 33.95	− 6.24	− 1.29	4.95
B_27_H_18_N_27_	− 15.94	− 8.05	1.08	9.12
B_26_H_18_N_27_Al	− 20.65	− 8.03	− 0.03	8.00

^a^The values are given in eV and *V*_min_ in kcal/mol.

#### MESP of circumcoronene and its analogues

**Figure 8:**
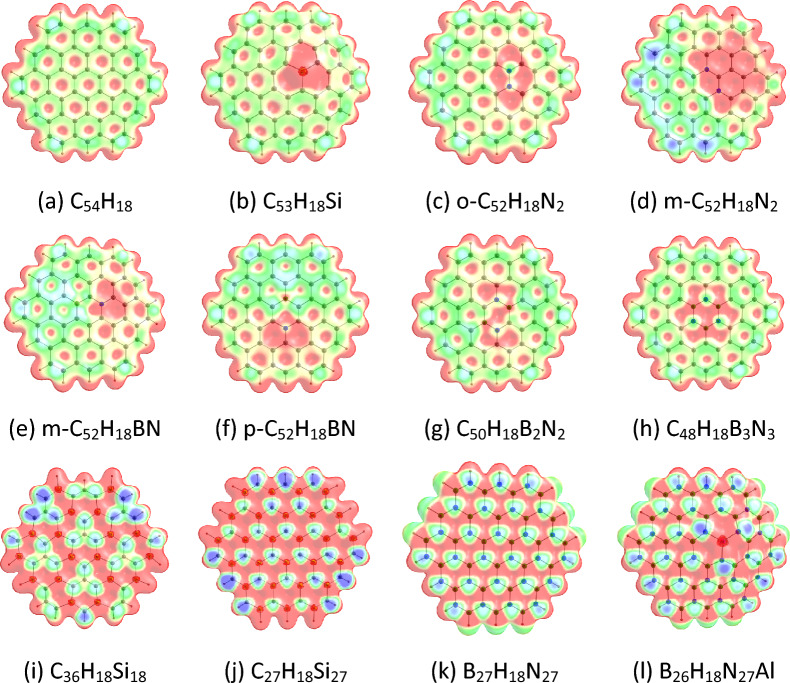
MESP mapped on 0.01 a.u. electron density isosurface of (**a**) C_54_H_18_, (**b**) C_53_H_18_Si, (**c**) o-C_52_H_18_N_2_, (**d**) m-C_52_H_18_N_2_, (**e**) m-C_52_H_18_BN, (**f**) p-C_52_H_18_BN, (**g**) C_50_H_18_B_2_N_2_, (**h**) C_48_H_18_B_3_N_3_, (**i**) C_36_H_18_Si_18_, (**j**) C_27_H_18_Si_27_, (**k**) B_27_H_18_N_27_, (**l**) B_26_H_18_N_27_Al. The Colour coding from blue to red indicates MESP values in the range -0.03 to 0.03 a.u. The Colours at the blue end of the spectrum indicate the electron-rich regions, while those toward the red indicate the electron-deficient regions.

The MESP features of circumcoronene and its analogues are given in Fig. 8. The MESP analysis indicate that as in the case of coronene, electron-rich regions (e.g., green regions) are present on circumcoronene. The replacements of C atoms of circumcoronene by Si/N/BN/AlN units lead to an increase of the electron-rich environments (e.g., blue regions) in the molecules. The MESP *V*_min_ values of circumcoronene and its analogues are provided in Table 4. The locations of *V*_min_ in the MESP plots of circumcoronene and its analogues are provided in Fig. S5. As in the case of coronene, the MESP *V*_min_ points are located near the peripheral rings of circumcoronene. For the doped circumcoronenes, the MESP *V*_min_ points are located mainly near the peripheral rings, except for C_53_H_18_Si, m-C_52_H_18_BN, p-C_52_H_18_BN, C_50_H_18_B_2_N_2_, and B_26_H_18_N_27_Al. We find that the MESP *V*_min_ values of circumcoronene and its analogues are in the range of − 14.56 (C_54_H_18_) to − 33.95 kcal/mol (C_27_H_18_Si_27_). The MESP *V*_min_ of the doped circumcoronene is higher than that of the pristine circumcoronene. For the N-doped circumcoronenes, the MESP *V*_min_ value of o-C_52_H_18_N_2_ (− 17.19 kcal/mol) is lower than that of m-C_52_H_18_N_2_ (-24.03 kcal/mol). The MESP *V*_min_ values of Si-doped circumcoronenes increase in the order C_53_H_18_Si < C_36_H_18_Si_18_ < C_27_H_18_Si_27_. Furthermore, the MESP *V*_min_ values of BN/AlN-doped circumcoronenes increase in the order C_48_H_18_B_3_N_3_ < B_27_H_18_N_27_ < C_50_H_18_B_2_N_2_ < B_26_H_18_N_27_Al < p-C_52_H_18_BN < m-C_52_H_18_BN.

#### Li^+^ adsorption on the doped circumcoronenes

**Figure 9:**
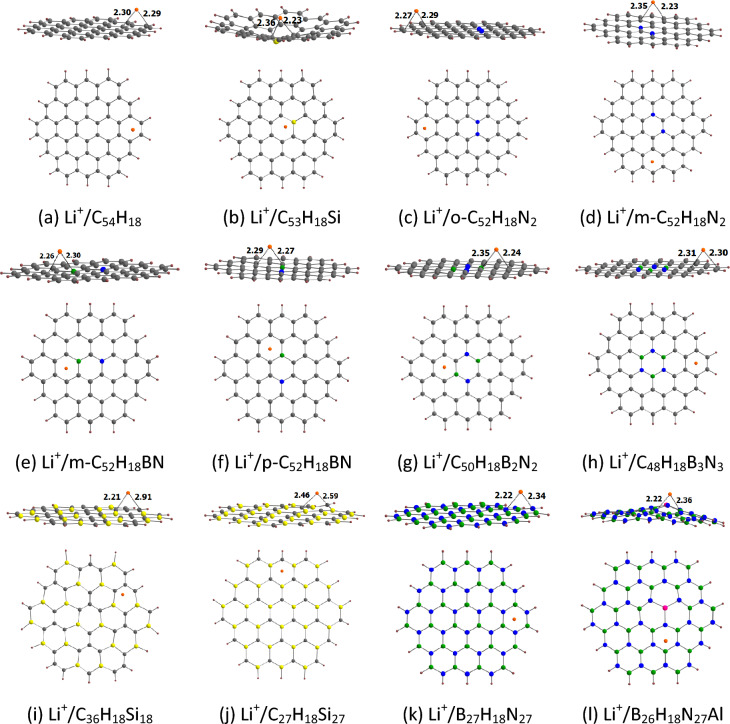
Optimized structures of Li^+^ adsorbed on (**a**) C_54_H_18_, (**b**) C_53_H_18_Si, (**c**) o-C_52_H_18_N_2_, (**d**) m-C_52_H_18_N_2_, (**e**) m-C_52_H_18_BN, (**f**) p-C_52_H_18_BN, (**g**) C_50_H_18_B_2_N_2_, (**h**) C_48_H_18_B_3_N_3_, (**i**) C_36_H_18_Si_18_, (**j**) C_27_H_18_Si_27_, (**k**) B_27_H_18_N_27_, (**l**) B_26_H_18_N_27_Al. The bond distances are given in Å. The color code is the same as in Fig. 4.

**Figure 10:**
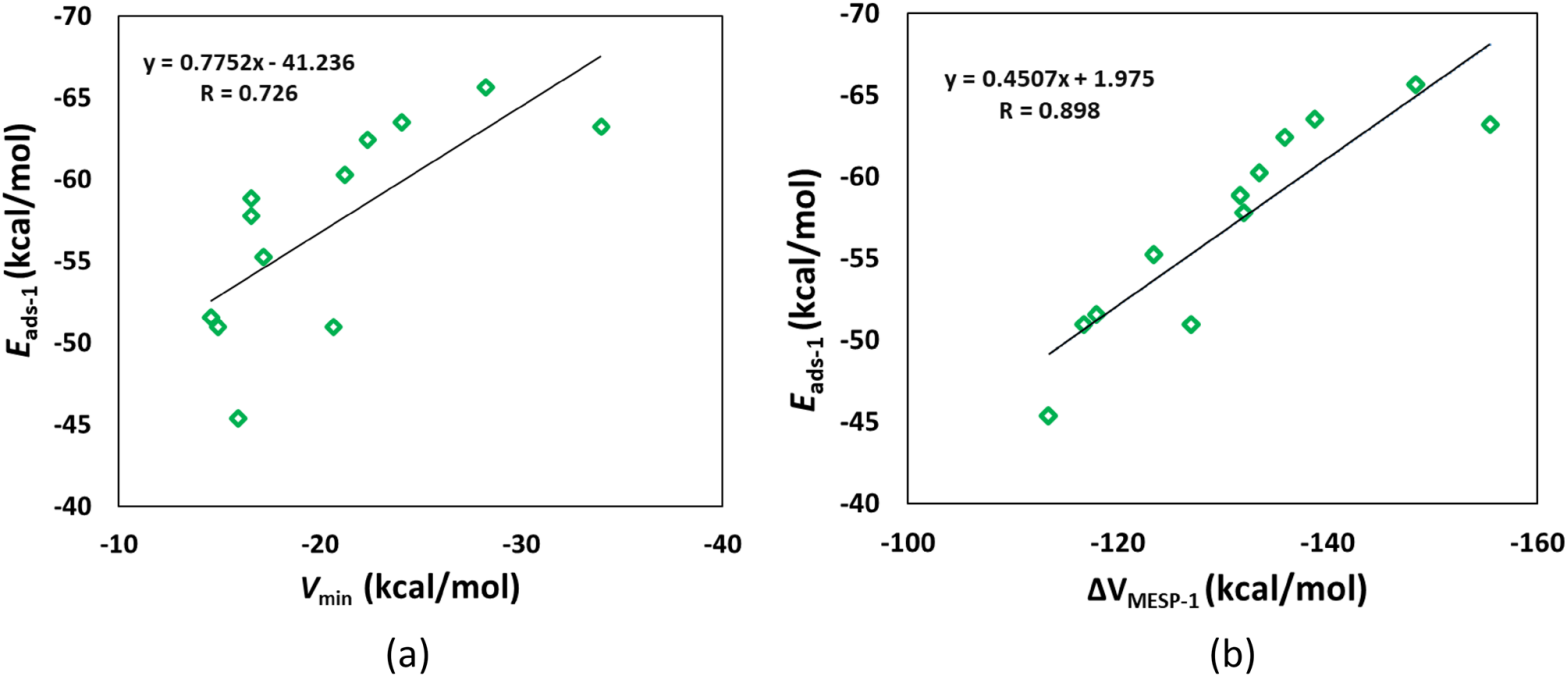
Correlation between (**a**) *V*_min_ and *E*_ads-1_ and (**b**) ∆*V*_MESP-1_ and *E*_ads-1_ for Li^+^-adsorbed circumcoronenes.

The optimized geometries for the adsorption of Li^+^ on circumcoronene and its analogues are provided in Fig. 9. Our results show that as in the case of coronene, Li^+^ binds to the peripheral rings of circumcoronene. For the doped circumcoronenes, Li^+^ binds to the peripheral rings, except for C_53_H_18_Si, m-C_52_H_18_BN, p-C_52_H_18_BN, C_50_H_18_B_2_N_2_, and B_26_H_18_N_27_Al. Our results show that Li^+^ is relatively strongly adsorbed on circumcoronene and its analogues. For example, the adsorption distance of Li^+^ on these adsorbents is in the range of 2.21 (C_36_H_18_Si_18_) to 2.46 Å (C_27_H_18_Si_27_). This observation is further supported by the adsorption energy of Li^+^ on circumcoronene and its analogues (Table 5). The E_ads-1_ values of Li^+^ on circumcoronene and its analogues are in the range of − 45.36 (B_27_H_18_N_27_) to − 65.61 kcal/mol (C_36_H_18_Si_18_). The more negative the E_ads-1_ value, the stronger the adsorption is. The E_ads-1_ value of Li^+^ on circumcoronene is − 51.55 kcal/mol. For the N-doped circumcoronenes, the E_ads-1_ value of o-C_52_H_18_N_2_ (− 55.24 kcal/mol) is higher than that of m-C_52_H_18_N_2_ (− 63.51 kcal/mol). The E_ads-1_ values of Si-doped circumcoronenes increase in the order C_53_H_18_Si < C_27_H_18_Si_27_ < C_36_H_18_Si_18_. Furthermore, the E_ads-1_ values of BN/AlN-doped circumcoronenes increase in the order B_27_H_18_N_27_ < C_48_H_18_B_3_N_3_ ≈ B_26_H_18_N_27_Al < C_50_H_18_B_2_N_2_ < p-C_52_H_18_BN < m-C_52_H_18_BN. These adsorption energies are correlated with the MESP *V*_min_ values of circumcoronene and its analogues, with a correlation coefficient of 0.726 (Fig. 10a). Note that a better correlation was observed for coronene and its analogues (see Fig. 5a).

**Table 5:** E_ads-1_, ∆*V*_MESP-1_, E_HOMO_ E_LUMO,_ E_g-2_ and ∆E_g-1_ values for Li^+^ adsorbed circumcoronenes^a^.

Nanoflake	E_ads-1_	∆*V*_MESP-1_	E_HOMO_	E_LUMO_	E_g-2_	∆E_g-1_
C_54_H_18_	− 51.55	− 117.95	− 8.40	− 4.37	4.04	− 7.3
C_53_H_18_Si	− 58.84	− 131.61	− 8.59	− 4.98	3.61	− 8.9
o-C_52_H_18_N_2_	− 55.24	− 123.41	− 7.17	− 4.26	2.92	− 4.2
m-C_52_H_18_N_2_	− 63.51	− 138.73	− 7.03	− 4.37	2.66	24.7
m-C_52_H_18_BN	− 62.42	− 135.94	− 8.40	− 4.55	3.84	− 2.0
p-C_52_H_18_BN	− 60.26	− 133.45	− 8.56	− 4.40	4.16	− 6.5
C_50_H_18_B_2_N_2_	− 57.77	− 131.99	− 8.26	− 4.74	3.52	3.0
C_48_H_18_B_3_N_3_	− 50.95	− 116.74	− 8.46	− 4.28	4.18	− 12.9
C_36_H_18_Si_18_	− 65.61	− 148.34	− 7.99	− 4.34	3.65	1.1
C_27_H_18_Si_27_	− 63.21	− 155.49	− 8.05	− 3.59	4.46	− 9.9
B_27_H_18_N_27_	− 45.36	− 113.38	− 10.10	− 4.09	6.01	− 34.1
B_26_H_18_N_27_Al	− 50.95	− 127.00	− 10.28	− 4.01	6.28	− 21.6

^a^The values of E_ads-1_ and Δ*V*_MESP-1_ in kcal/mol; E_HOMO_, E_LUMO_, and E_g-2_ in eV; ∆E_g-1_ in %.

The negative values of Δ*V*_MESP-1_ (Table 5) indicate the electron donation from circumcoronene and its analogues to Li^+^. These Δ*V*_MESP-1_ values are in the range of − 113.38 (B_27_H_18_N_27_) to − 155.49 kcal/mol (C_27_H_18_Si_27_). The Δ*V*_MESP-1_ value of circumcoronene is − 117.95 kcal/mol. The Δ*V*_MESP-1_ of the doped circumcoronenes is typically higher than that of the pristine circumcoronene. However, the Δ*V*_MESP-1_ of C_48_H_18_B_3_N_3_ and B_12_H_12_N_12_ is lower than that of circumcoronene. The adsorption energies of Li^+^ on circumcoronene and its analogues are correlated with these Δ*V*_MESP-1_ values, with a correlation coefficient of 0.898 (Fig. 10b). This behavior is similar to what we observe for coronene and its analogues (see Fig. 5b).

The E_HOMO,_ E_LUMO_, and E_g-2_ values of Li^+^-adsorbed circumcoronene and its analogues are given in Table 5. The E_HOMO,_ E_LUMO_, and E_g-2_ values of Li^+^-adsorbed circumcoronene and its analogues are in the ranges of − 10.28 (B_26_H_18_N_27_Al) to − 7.03 eV (m-C_52_H_18_N_2_), − 4.98 (C_53_H_18_Si) to − 3.59 eV (C_27_H_18_Si_27_), and 2.66 (m-C_52_H_18_N_2_) to 6.28 eV (B_26_H_18_N_27_Al), respectively. Here also, E_HOMO_ of the Li^+^-adsorbed nanoflakes is lower than that of the pristine nanoflakes (see Table 4) for all cases. A similar result is obtained for E_LUMO_. Our results here show that E_g-2_ is typically lower than E_g-1_. For example, the E_HOMO_, E_LUMO_, and E_g-2_ values of Li^+^-adsorbed circumcoronene are − 8.40, − 4.37, and 4.04 eV, respectively. The decrease in the HOMO–LUMO energy gap for the adsorption of Li^+^ on circumcoronene is 7.3% (see ΔE_g-1_ values in Table 5). A maximum decrease in the HOMO–LUMO energy gap of 34.1% was observed for the adsorption of Li^+^ on B_27_H_18_N_27_. However, E_g-2_ is found to be higher than E_g-1_ for the adsorption of Li^+^ on m-C_52_H_18_N_2_, C_50_H_18_B_2_N_2_, and C_36_H_18_Si_18_. A maximum increase in the HOMO–LUMO energy gap of 24.7% was observed for the adsorption of Li^+^ on m-C_52_H_18_N_2_.

#### Li adsorption on the doped circumcoronenes

**Figure 11:**
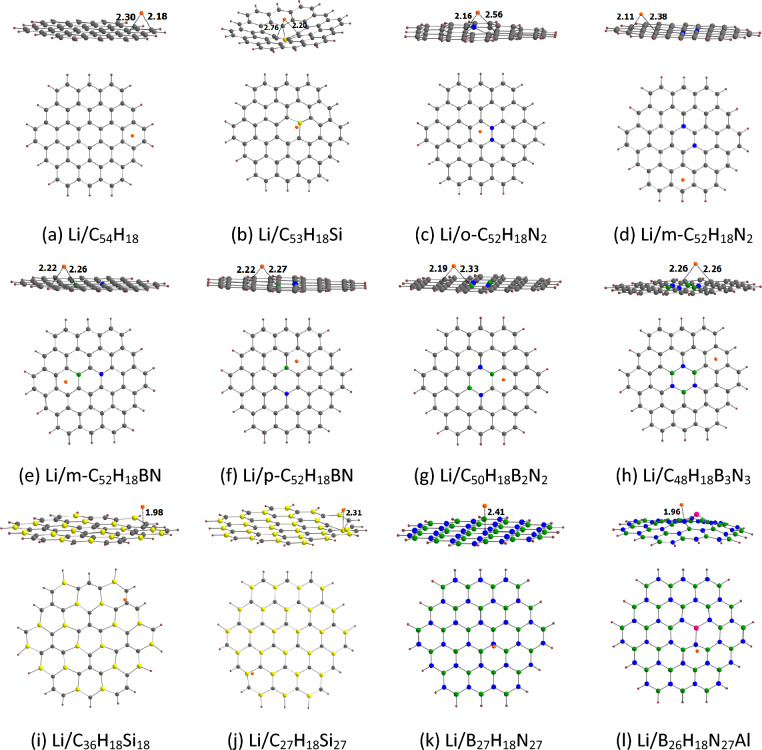
Optimized structures of Li adsorbed on (**a**) C_54_H_18_, (**b**) C_53_H_18_Si, (**c**) o-C_52_H_18_N_2_, (**d**) m-C_52_H_18_N_2_, (**e**) m-C_52_H_18_BN, (**f**) p-C_52_H_18_BN, (**g**) C_50_H_18_B_2_N_2_, (**h**) C_48_H_18_B_3_N_3_, (**i**) C_36_H_18_Si_18_, (**j**) C_27_H_18_Si_27_, (**k**) B_27_H_18_N_27_, (**l**) B_26_H_18_N_27_Al. The bond distances are given in Å. The color code is the same as in Fig. 4.

The optimized geometries for the adsorption of Li on circumcoronene and its analogues are provided in Fig. 11. Our results show that Li binds to the peripheral rings of circumcoronene, similar to the results obtained for Li^+^. For the doped circumcoronenes, Li binds to the peripheral rings only of m-C_52_H_18_N_2_, C_48_H_18_B_3_N_3_, C_36_H_18_Si_18_, and C_27_H_18_Si_27_. The adsorption distance of Li on these adsorbents is in the range of 1.96 (B_26_H_18_N_27_Al) to 2.41 Å (B_27_H_18_N_27_). For these adsorbents, the adsorption distance of Li is generally smaller than that of Li^+^ (see Fig. 10). For example, the adsorption distance of Li and Li^+^ on circumcoronene is 2.18 and 2.29 Å, respectively. However, as in the case of coronene and its analogues, Li is weakly adsorbed on circumcoronene and its analogues when compared to Li^+^. This observation is supported by the adsorption energy of Li on circumcoronene and its analogues (Table 6). The E_ads-2_ values of Li on circumcoronene and its analogues are in the range of − 3.07 (B_27_H_18_N_27_) to − 47.79 kcal/mol (C_53_H_18_Si). The E_ads-2_ value of Li on circumcoronene is − 18.73kcal/mol. For the N-doped circumcoronenes, the E_ads-2_ value of o-C_52_H_18_N_2_ (− 22.71 kcal/mol) is lower than that of m-C_52_H_18_N_2_ (− 32.31 kcal/mol). The E_ads-2_ values of Si-doped circumcoronenes increase in the order C_27_H_18_Si_27_ < C_36_H_18_Si_18_ < C_53_H_18_Si. Furthermore, the E_ads-2_ values of BN/AlN-doped circumcoronenes increase in the order B_27_H_18_N_27_ < C_48_H_18_B_3_N_3_ < B_26_H_18_N_27_Al < p-C_52_H_18_BN < m-C_52_H_18_BN < C_50_H_18_B_2_N_2_. These adsorption energies are not well correlated with the MESP *V*_min_ values of circumcoronene and its analogues (Fig. S6).

**Table 6:** E_ads-2_, ∆*V*_MESP-2_, E_HOMO_ E_LUMO,_ E_g-3_ and ∆E_g-2_ values for Li adsorbed circumcoronenes, along with ∆E_cell_ and V_cell_^a^.

Nanoflake	E_ads-2_	∆*V*_MESP-2_	E_HOMO_	E_LUMO_	E_g-3_	∆E_g-2_	∆E_cell_	V_cell_
C_54_H_18_	− 18.73	29.30	− 3.63	− 1.43	2.21	− 49.3	− 32.82	1.42
C_53_H_18_Si	− 47.79	8.95	− 4.68	− 1.57	3.11	− 21.4	− 11.06	0.48
o-C_52_H_18_N_2_	− 22.71	23.12	− 3.89	− 1.17	2.72	− 10.5	− 32.53	1.41
m-C_52_H_18_N_2_	− 32.31	24.13	− 3.82	− 1.18	2.64	23.8	− 31.19	1.35
m-C_52_H_18_BN	− 33.72	18.56	− 3.81	− 1.38	2.42	− 38.2	− 28.69	1.24
p-C_52_H_18_BN	− 27.78	19.14	− 3.64	− 1.45	2.20	− 50.7	− 32.48	1.41
C_50_H_18_B_2_N_2_	− 34.06	24.52	− 4.04	− 1.31	2.72	− 20.2	− 23.71	1.03
C_48_H_18_B_3_N_3_	− 20.93	20.12	− 3.80	− 1.20	2.61	− 45.8	− 30.02	1.30
C_36_H_18_Si_18_	− 36.84	3.49	− 4.21	− 1.69	2.52	− 30.2	− 28.78	1.25
C_27_H_18_Si_27_	− 27.70	− 15.89	− 4.84	− 1.26	3.58	− 27.7	− 35.52	1.54
B_27_H_18_N_27_	− 3.07	− 22.14	− 3.21	0.53	3.74	− 59.0	− 42.29	1.83
B_26_H_18_N_27_Al	− 23.35	17.35	− 5.22	− 0.77	4.44	− 44.4	− 27.60	1.20

^a^The values of E_ads-2_, Δ*V*_MESP-2_, and ∆E_cell_ in kcal/mol; E_HOMO_, E_LUMO_, and E_g-3_ in eV; ∆E_g-2_ in %; V_cell_ in V.

In general, the values of Δ*V*_MESP-2_ are positive for the adsorption of Li on circumcoronene and its analogues (Table 6). The positive value of Δ*V*_MESP-2_ suggests the electron density transfer from Li to nanoflakes. However, the values of Δ*V*_MESP-2_ are negative for the adsorption of Li on C_27_H_18_Si_27_ and B_27_H_18_N_27_. Here also, E_ads-2_ does not show a correlation with Δ*V*_MESP-2_ (see Fig. S6).

The E_HOMO,_ E_LUMO_, and E_g-3_ values of Li-adsorbed circumcoronene and its analogues are given in Table 6. The E_HOMO,_ E_LUMO_, and E_g-3_ values of Li-adsorbed circumcoronene and its analogues are in the ranges of − 5.22 (B_26_H_18_N_27_Al) to − 3.21 eV (B_27_H_18_N_27_), − 1.69 (C_36_H_18_Si_18_) to 0.53 eV (B_27_H_18_N_27_), and 2.20 (p-C_52_H_18_BN) to 4.44 eV (B_26_H_18_N_27_Al), respectively. Here also, E_HOMO_ of the Li-adsorbed nanoflakes is higher than that of the pristine nanoflakes (see Table 4) for all cases. For example, the E_HOMO_ value of Li-adsorbed circumcoronene is − 3.63 eV. The E_LUMO_ of circumcoronene and its analogues is not much affected by the presence of Li. The results here show that E_g-3_ is usually lower than E_g-1_. The decrease in the HOMO–LUMO energy gap for the adsorption of Li on circumcoronene is 49.3% (see ΔE_g-2_ values in Table 3). A maximum decrease in the HOMO–LUMO energy gap of 59.0% was observed for the adsorption of Li on B_27_H_18_N_27_. However, E_g-3_ is found to be higher than E_g-1_ for the adsorption of Li on m-C_52_H_18_N_2_ (ΔE_g-2_ of 23.8%).

#### Cell voltage of circumcoronene and its analogues

The ΔE_cell_ and V_cell_ of LIBs based on circumcoronene and its analogues are given in Table 6. Here the ∆E_cell_ and V_cell_ values are in the ranges of − 42.29 (B_27_H_18_N_27_) to − 11.06 kcal/mol (C_53_H_18_Si) and 0.48 (C_53_H_18_Si) to 1.83 V (B_27_H_18_N_27_), respectively. As mentioned before, the nanoflakes interacting strongly with Li^+^ and weakly with Li may serve as good candidates for the anode materials in LiBs. For example, the highest V_cell_ value of 1.83 V is observed for B_27_H_18_N_27_ due to the high E_ads-1_ of − 45.36 kcal/mol (see Table 5) and low E_ads-2_ of − 3.07 kcal/mol (see Table 6). The lowest V_cell_ value of 0.48 V observed for C_53_H_18_Si can be attributed to a higher contribution from E_ads-2_ (E_ads-1_ of − 58.84 kcal/mol and E_ads-2_ of − 47.79 kcal/mol). The ∆E_cell_ and V_cell_ values of circumcoronene are − 32.82 kcal/mol and 1.42 V, respectively. For the N-doped circumcoronenes, the V_cell_ value of o-C_52_H_18_N_2_ (1.41 V) is higher than that of o-C_52_H_18_N_2_ (1.35 V). The V_cell_ values of Si-doped circumcoronenes increase in the order C_53_H_18_Si < C_36_H_18_Si_18_ < C_27_H_18_Si_27_. Furthermore, the V_cell_ values of BN/AlN-doped circumcoronenes increase in the order C_50_H_18_B_2_N_2_ < B_26_H_18_N_27_Al < m-C_52_H_18_BN < C_48_H_18_B_3_N_3_ < p-C_52_H_18_BN < B_27_H_18_N_27_. Here also, the linear correlation of E_ads-2_ with V_cell_ (R = 0.840) supports the strong influence of E_ads-2_ on V_cell_ (see Fig. S4).

The adsorption free energies (Δ*G*_ads_ = G_adsorbate/nanoflake_ – (G_nanoflake_ + G_adsorbate_)) show that the adsorption of Li^+^ on the nanoflakes is exergonic (Table S1). The Δ*G*_ads_ values for the adsorption of Li^+^ on the nanoflakes are in the range of − 33.69 (B_12_H_12_N_12_) to − 56.84 kcal/mol (C_36_H_18_Si_18_). The adsorption of Li on the nanoflakes is generally exergonic. However, the adsorption of Li on B_12_H_12_N_12_ and B_27_H_18_N_27_ is endergonic, indicating weak interactions.

The storage capacity (*C* = *(n x F* × *10*^*3*^*)/M,* where *n* is the maximum number of adsorbed Li atoms and *M* is the molecular mass of the electrode)^43^ is an important factor for the performance of LIBs. To obtain the lithium storage capacity, we examined the interactions of Li atoms with, for example, C_24_H_12_. The resulting 6Li@C_24_H_12_ (Fig. S7) is found to be a true local minimum with no imaginary frequency. Therefore, the lithium storage capacity of C_24_H_12_ is 536.2 mAh/g, indicating that such nanoflakes are promising for high-capacity LIBs. For a comparison, the lithium storage capacity of graphite^44^ is 372 mAh/g, Sc_2_C MXene^45^ is 462 mAh/g, graphene-like C_2_N^46^ is 671.7 mAh/g, and pentagraphyne^47^ is 687 mAh/g.

There have been experimental studies on the use of PAHs in LIBs. For example, Park et al. obtained excellent rate capability and cycle endurance for LIBs using PAHs as anode materials^9^. There have also been DFT studies on the potential use of PAHs in LIBs. For example, based on the cell voltage data, Remya and Suresh suggested coronene as a good anode material for LIBs^27^. Based on our *V*_cell_ data, C_24_H_12_, C_12_H_12_Si_12_, B_12_H_12_N_12_, C_27_H_18_Si_27_, and B_27_H_18_N_27_ are suggested as good anode materials for the LIBs and B_27_H_18_N_27_ is the best among all tested nanoflakes. The *V*_cell_ of B_27_H_18_N_27_ is found to be 1.83 V. For a comparison, the *V*_cell_ of circumbiphenyl is 1.51 V^27^, sumanene is 1.68 V^48^, and hexa-peri-hexabenzocoronene is 1.70 V^49^. The insights on the adsorption mechanism from this study could help design an efficient electrode which could be useful for the further development efficient LIBs.

## Conclusions

We performed DFT calculations to investigate the adsorption mechanisms of Li^+^ and Li on a total of twenty-four adsorbents (C_24_H_12_ and its analogues C_23_H_12_Si, o-C_22_H_12_N_2_, m-C_22_H_12_N_2_, m-C_22_H_12_BN, p-C_22_H_12_BN, C_20_H_12_B_2_N_2_, C_18_H_12_B_3_N_3_, C_16_H_12_Si_8_, C_12_H_12_Si_12_, B_12_H_12_N_12_, B_11_H_12_N_12_Al; C_54_H_18_ and its analogues C_53_H_18_Si, o-C_52_H_18_N_2_, m-C_52_H_18_N_2_, m-C_52_H_18_BN, p-C_52_H_18_BN, C_50_H_18_B_2_N_2_, C_48_H_18_B_3_N_3_, C_36_H_18_Si_18_, C_27_H_18_Si_27_, B_27_H_18_N_27_, B_26_H_18_N_27_Al). The XY (X = C, Si, N, B, Al; Y = C, Si, N, B, Al) bond lengths were in the range of about 1.35 to 1.81 Å for all adsorbents. Here all the CSi and NAl bond lengths were found to be significantly longer than all the CC bond lengths. The MESP analysis show that the replacements of C atoms of coronene and circumcoronene by Si/N/BN/AlN units lead to an increase of the electron-rich environments in the molecules.

Li^+^ is relatively strongly adsorbed on all adsorbents. For example, the adsorption distance of Li^+^ on all adsorbents is in the range of 2.15 (C_16_H_12_Si_8_) to 2.46 Å (C_27_H_18_Si_27_). This observation is further supported by the adsorption energy of Li^+^ on the nanoflakes. The E_ads-1_ values of Li^+^ on all adsorbents are in the range of − 42.47 (B_12_H_12_N_12_) to − 66.26 kcal/mol (m-C_22_H_12_BN). Our results indicate a stronger interaction between Li^+^ and the nanoflakes as the MESP *V*_min_ of the nanoflakes becomes more negative. The electron donation from the nanoflakes to Li^+^ could be checked by assessing the changes in the MESP at the nucleus of Li^+^ upon adsorption. Our results show that a stronger interaction between Li^+^ and the nanoflakes pushes more electron density toward Li^+^. The HOMO–LUMO gap of Li^+^-adsorbed nanoflakes is typically lower than that of pristine ones.

The adsorption distance of Li on the nanoflakes is generally smaller than that of Li^+^. For example, the adsorption distance of Li and Li^+^ on circumcoronene is 2.18 and 2.29 Å, respectively. However, Li is weakly adsorbed on circumcoronene and its analogues when compared to Li^+^. This observation is supported by the adsorption energy of Li on the nanoflakes. The E_ads-2_ values of Li on all adsorbents are in the range of − 3.07 (B_27_H_18_N_27_) to − 47.79 kcal/mol (C_53_H_18_Si). The HOMO–LUMO gap of Li-adsorbed nanoflakes is typically lower than that of pristine ones. The nanoflakes interacting strongly with Li^+^ and weakly with Li may serve as good candidates for the anode materials in LiBs. Among all adsorbents, the highest V_cell_ value of 1.83 V is observed for B_27_H_18_N_27_ due to the high E_ads-1_ of − 45.36 kcal/mol and low E_ads-2_ of − 3.07 kcal/mol. The E_ads-1_ data of studied systems show only a small variation compared to E_ads-2_, and as a result, E_ads-2_ has a relatively strong influence on the variation of V_cell_.

### Supplementary Information

Supplementary Information.

## Data Availability

All data generated or analyzed during this study are included in the article and supporting information.

## References

[1] Whittingham MS (2004). Lithium batteries and cathode materials. Chem. Rev..

[2] Blomgren GE (2017). The development and future of lithium ion batteries. J. Electrochem. Soc..

[3] Ruiz V (2018). A review of international abuse testing standards and regulations for lithium ion batteries in electric and hybrid electric vehicles. Renew. Sustain. Energy Rev..

[4] Yuan W (2016). The applications of carbon nanotubes and graphene in advanced rechargeable lithium batteries. J. Mater. Chem. A.

[5] Raccichini R, Varzi A, Passerini S, Scrosati B (2015). The role of graphene for electrochemical energy storage. Nat. Mater..

[6] Huang X (2011). Graphene-based materials: Synthesis, characterization, properties, and applications. Small.

[7] Li W, Li M, Adair KR, Sun X, Yu Y (2017). Carbon nanofiber-based nanostructures for lithium-ion and sodium-ion batteries. J. Mater. Chem. A.

[8] Chang S (2022). In situ formation of polycyclic aromatic hydrocarbons as an artificial hybrid layer for lithium metal anodes. Nano Lett..

[9] Park J (2018). Contorted polycyclic aromatic hydrocarbon: Promising Li insertion organic anode. J. Mater. Chem. A.

[10] Wu Z-S, Ren W, Xu L, Li F, Cheng H-M (2011). Doped graphene sheets as anode materials with superhigh rate and large capacity for lithium ion batteries. ACS Nano.

[11] Bulusheva LG (2011). Electrochemical properties of nitrogen-doped carbon nanotube anode in Li-ion batteries. Carbon.

[12] Yue H (2014). Nitrogen-doped carbon nanofibers as anode material for high-capacity and binder-free lithium ion battery. Mater. Lett..

[13] Zhang L (2016). Boron and nitrogen co-doped porous carbon nanotubes webs as a high-performance anode material for lithium ion batteries. Int. J. Hydrogen Energy..

[14] Aghamohammadi H, Hassanzadeh N, Eslami-Farsani R (2021). A review study on the recent advances in developing the heteroatom-doped graphene and porous graphene as superior anode materials for Li-ion batteries. Ceram. Int..

[15] Stępień M, Gońka E, Żyła M, Sprutta N (2017). Heterocyclic nanographenes and other polycyclic heteroaromatic compounds: Synthetic routes, properties, and applications. Chem. Rev..

[16] Jiang Z (2022). Synthesis, structure, and photophysical properties of BN-embedded analogue of coronene. Org. Lett..

[17] Zou Y (2023). Circumcoronenes. Angew. Chem. Int. Ed. Engl..

[18] Galano A (2007). Influence of silicon defects on the adsorption of thiophene-like compounds on polycyclic aromatic hydrocarbons: A theoretical study using thiophene + coronene as the simplest model. J. Phys. Chem. A.

[19] Ghatee MH, Moosavi F (2011). Physisorption of hydrophobic and hydrophilic 1-alkyl-3-methylimidazolium ionic liquids on the graphenes. J. Phys. Chem. C.

[20] Shakourian-Fard M, Kamath G, Jamshidi Z (2014). Trends in physisorption of ionic liquids on boron-nitride sheets. J. Phys. Chem. C.

[21] Malček M, Cordeiro MNDS (2018). A DFT and QTAIM study of the adsorption of organic molecules over the copper-doped coronene and circumcoronene. Phys. E Low-Dimens. Syst. Nanostructures.

[22] Velázquez-López LF, Pacheco-Ortin SM, Mejía-Olvera R, Agacino-Valdés E (2019). DFT study of CO adsorption on nitrogen/boron doped-graphene for sensor applications. J. Mol. Model..

[23] Hussain R (2020). Density functional theory study of palladium cluster adsorption on a graphene support. RSC Adv..

[24] dos Santos TC (2021). CO_2_ and H_2_ adsorption on 3D nitrogen-doped porous graphene: Experimental and theoretical studies. J. CO2 Util..

[25] Gal J-F (2003). Lithium-cation/π complexes of aromatic systems. The effect of increasing the number of fused rings. J. Am. Chem. Soc..

[26] Pattarapongdilok N, Parasuk V (2020). Adsorptions of lithium ion/atom and packing of Li ions on graphene quantum dots: Application for Li-ion battery. Comput. Theor. Chem..

[27] Ramya PK, Suresh CH (2023). Polycyclic aromatic hydrocarbons as anode materials in lithium-ion batteries: A DFT study. J. Phys. Chem. A.

[28] Denis PA, Ullah S, Iribarne F (2020). Reduction chemistry of hexagonal boron nitride sheets and graphene: A comparative study on the effect of alkali atom doping on their chemical reactivity. New J. Chem..

[29] Saha B, Bhattacharyya PK (2019). Anion⋯π interaction in oxoanion-graphene complex using coronene as model system: A DFT study. Comput. Theor. Chem..

[30] Khudhair AM, Ben Ahmed A (2023). Utilizing circumcoronene and BN circumcoronene for the delivery and adsorption of the anticancer drug floxuridine. Comput. Theor. Chem..

[31] Geetha Sadasivan Nair R, Narayanan Nair AK, Sun S (2023). Adsorption of hazardous gases on Cyclo[18]carbon and its analogues. J. Mol. Liq..

[32] Geetha Sadasivan Nair R, Narayanan Nair AK, Sun S (2023). Adsorption of Gases on Fullerene-like X_12_Y_12_ (X = Be, Mg, Ca, B, Al, Ga, C; Y = C, Si, N, P, O) Nanocages. Energy Fuels.

[33] Frisch, M. J. *et al.* (Gaussian, Inc. 2016).

[34] Zhao Y, Truhlar DG (2008). The M06 suite of density functionals for main group thermochemistry, thermochemical kinetics, noncovalent interactions, excited states, and transition elements: Two new functionals and systematic testing of four M06-class functionals and 12 other functionals. Theor. Chem. Acc..

[35] Suresh CH, Remya GS, Anjalikrishna PK (2022). Molecular electrostatic potential analysis: A powerful tool to interpret and predict chemical reactivity. WIREs Comput. Mol. Sci..

[36] Remya GS, Suresh CH (2018). Assessment of the electron donor properties of substituted phenanthroline ligands in molybdenum carbonyl complexes using molecular electrostatic potentials. New J. Chem..

[37] Remya GS, Suresh CH (2019). Hydrogen elimination reactivity of ruthenium pincer hydride complexes: A DFT study. New J. Chem..

[38] Boys SF, Bernardi F (1970). The calculation of small molecular interactions by the differences of separate total energies. Some procedures with reduced errors. Mol. Phys..

[39] Yu Y-X (2016). Prediction of mobility, enhanced storage capacity, and volume change during sodiation on interlayer-expanded functionalized Ti_3_C_2_ MXene anode materials for sodium-ion batteries. J. Phys. Chem. C.

[40] Mittendorfer F, Hafner J (2001). Density-functional study of the adsorption of benzene on the (111), (100) and (110) surfaces of nickel. Surf. Sci..

[41] Weinelt M (1992). The electronic structure of ethylene on Ni(110): An experimental and theoretical study. Surf. Sci..

[42] Sayyed FB, Suresh CH (2011). Quantitative assessment of substituent effects on cation−π interactions using molecular electrostatic potential topography. J. Phys. Chem. A.

[43] Shakerzadeh E, Azizinia L (2021). Can C_24_N_24_ cavernous nitride fullerene be a potential anode material for Li-, Na-, K-, Mg-, Ca-ion batteries?. Chem. Phys. Lett..

[44] Dahn JR, Zheng T, Liu Y, Xue JS (1995). Mechanisms for lithium insertion in carbonaceous materials. Science.

[45] Lv X (2017). Sc2C as a promising anode material with high mobility and capacity: A first-principles study. ChemPhysChem.

[46] Zhang J (2019). Graphene-like carbon-nitrogen materials as anode materials for Li-ion and mg-ion batteries. Appl. Surf. Sci..

[47] Deb J, Ahuja R, Sarkar U (2022). Two-dimensional pentagraphyne as a high-performance anode material for Li/Na-ion rechargeable batteries. ACS Appl. Nano Mater..

[48] Gharibzadeh F, Vessally E, Edjlali L, Eshaghi M, Mohammadi R (2020). A DFT study on sumanene, corannulene, and nanosheet as the anodes in Li−ion batteries. Iran. J. Chem. Chem. Eng..

[49] Wu X, Zhang Z, Soleymanabadi H (2020). Substituent effect on the cell voltage of nanographene based Li-ion batteries: A DFT study. Solid State Commun..

