# Supplementary material for: Density functional theory study of doped coronene and circumcoronene as anode materials in lithium-ion batteries
Source: Sci Rep. 2024 Jul 2;14:15220. doi: 10.1038/s41598-024-66099-6 (PMC11219892; doi:10.1038/s41598-024-66099-6)
Supplement: Supplementary file 1 — Supplementary Information. [file 41598_2024_66099_MOESM1_ESM.docx]

***Supporting Information***

**Density Functional Theory Study of Doped Coronene and Circumcoronene as Anode Materials in Lithium-Ion Batteries**

Remya Geetha Sadasivan Nair^∗^, Arun Kumar Narayanan Nair^∗^, and Shuyu Sun^∗^

Physical Science and Engineering Division (PSE), Computational Transport Phenomena Laboratory, King Abdullah University of Science and Technology (KAUST), Thuwal, 23955-6900, Saudi Arabia.

* To whom correspondence should be addressed,

email: [remya.nair@kaust.edu.sa](mailto:remya.nair@kaust.edu.sa); [arun.narayanannair@kaust.edu.sa](mailto:arun.narayanannair@kaust.edu.sa); [shuyu.sun@kaust.edu.sa](mailto:shuyu.sun@kaust.edu.sa)

| 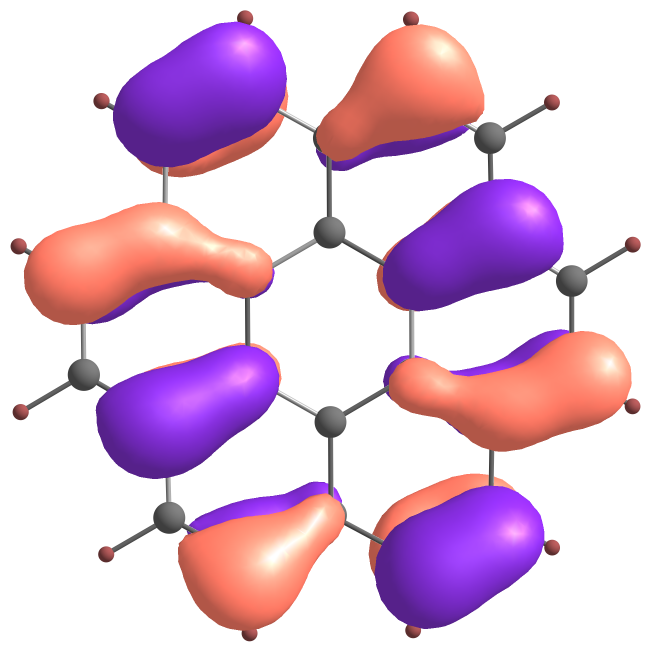 | 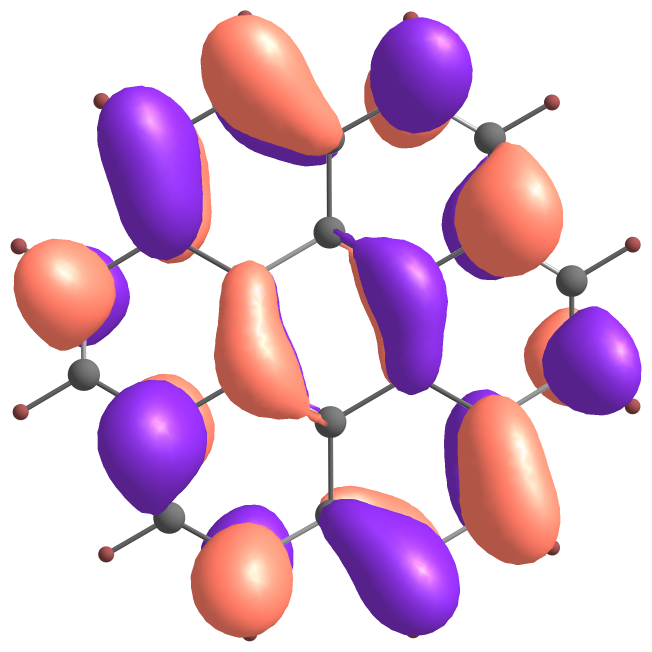 | 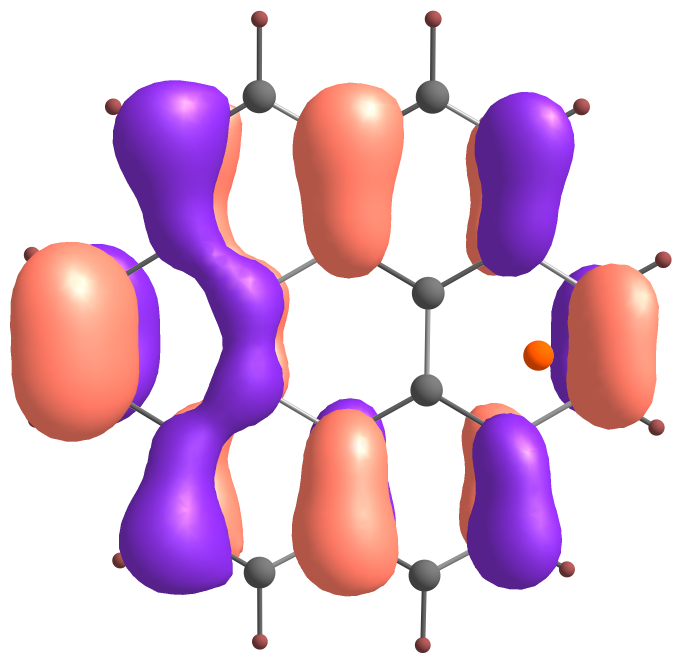 | 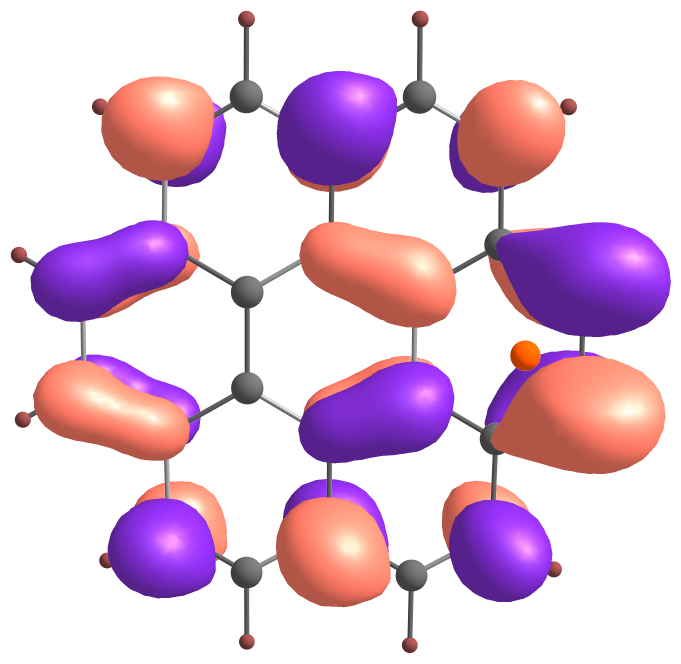 | 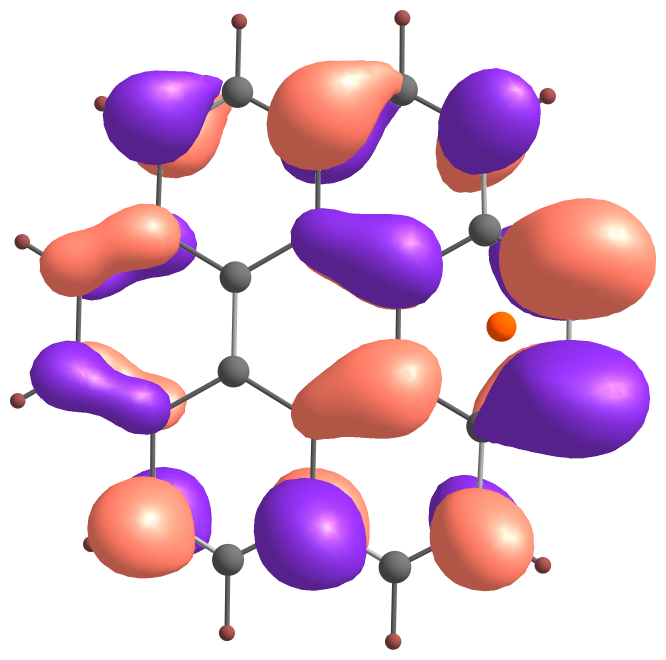 | 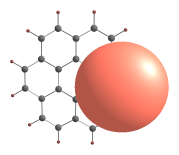 |
| --- | --- | --- | --- | --- | --- |
| C_24_H_12_  HOMO | C_24_H_12_  LUMO | Li^+^/C_24_H_12_  HOMO | Li^+^/C_24_H_12_  LUMO | Li/C_24_H_12_  HOMO | Li/C_24_H_12_  LUMO |
| 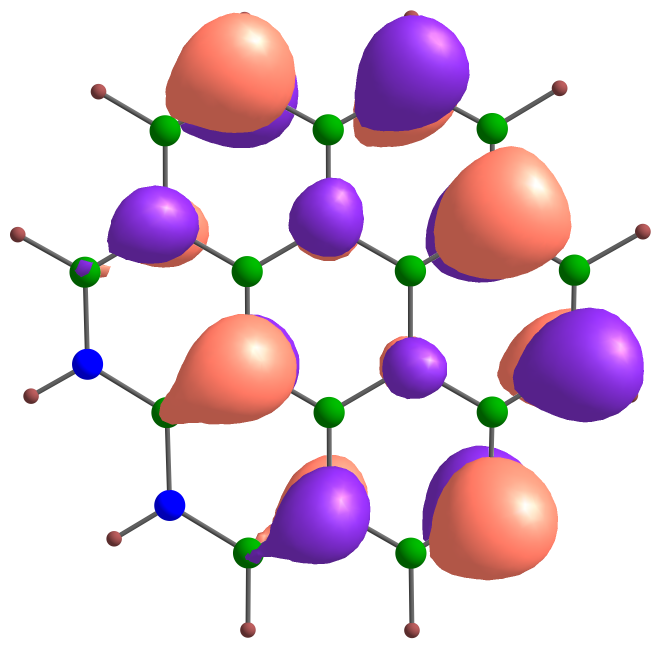 | 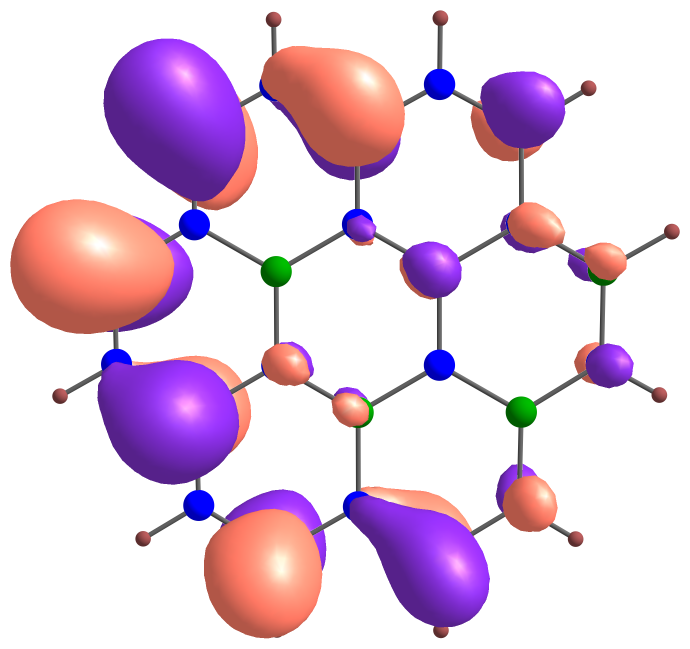 | 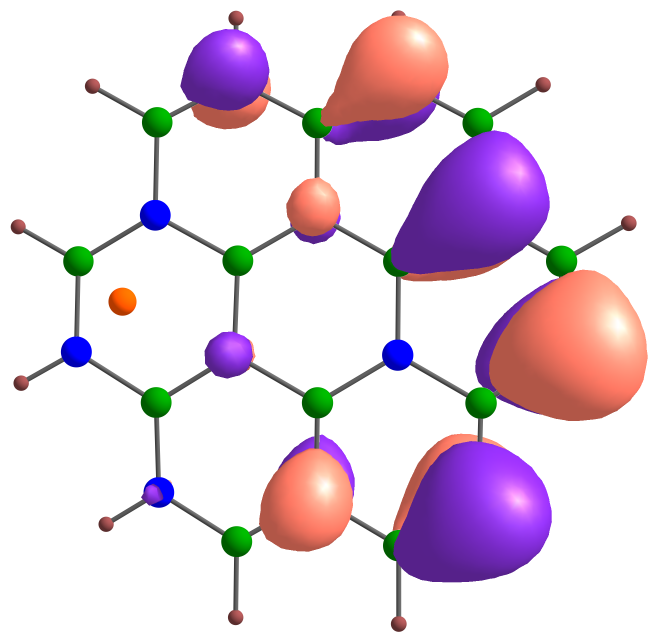 | 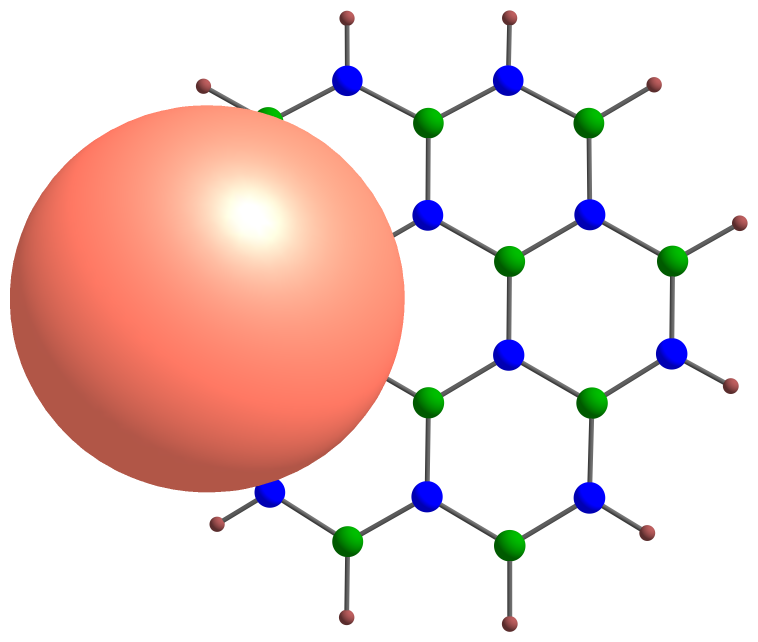 | 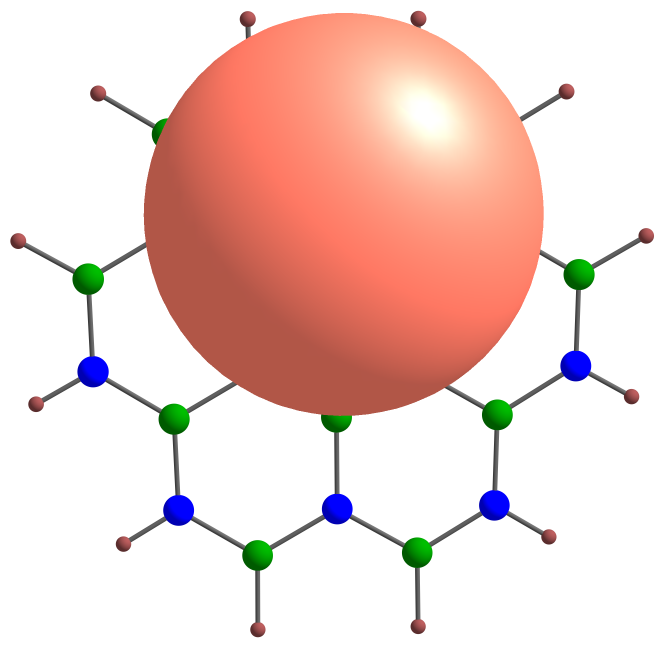 | 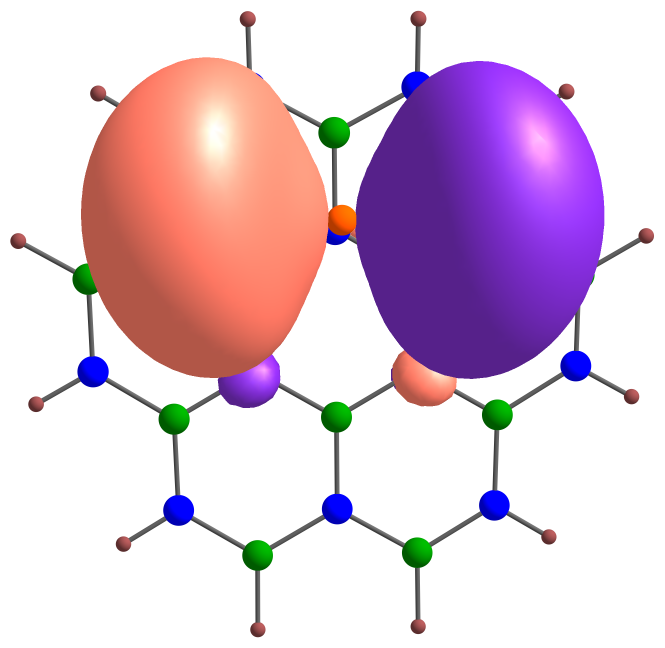 |
| B_12_H_12_N_12_  HOMO | B_12_H_12_N_12_  LUMO | Li^+^/B_12_H_12_N_12_  HOMO | Li^+^/B_12_H_12_N_12_  LUMO | Li/B_12_H_12_N_12_  HOMO | Li/B_12_H_12_N_12_  LUMO |
| 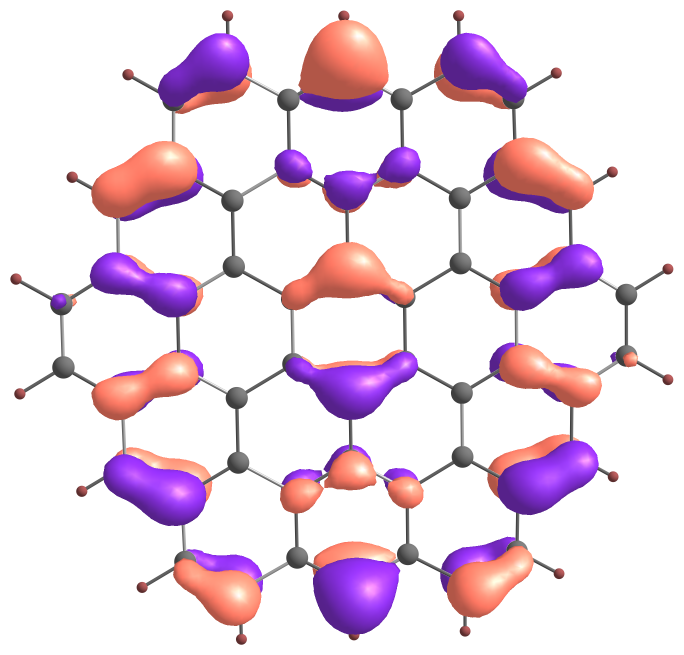 | 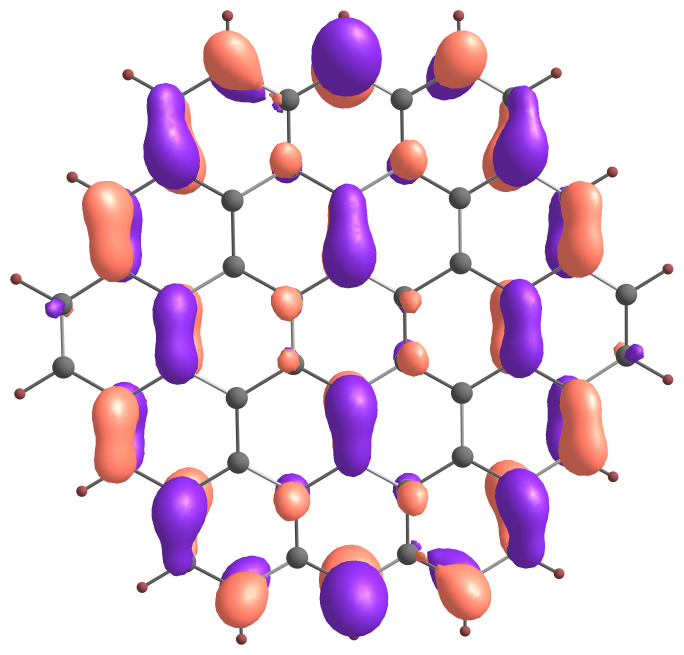 | 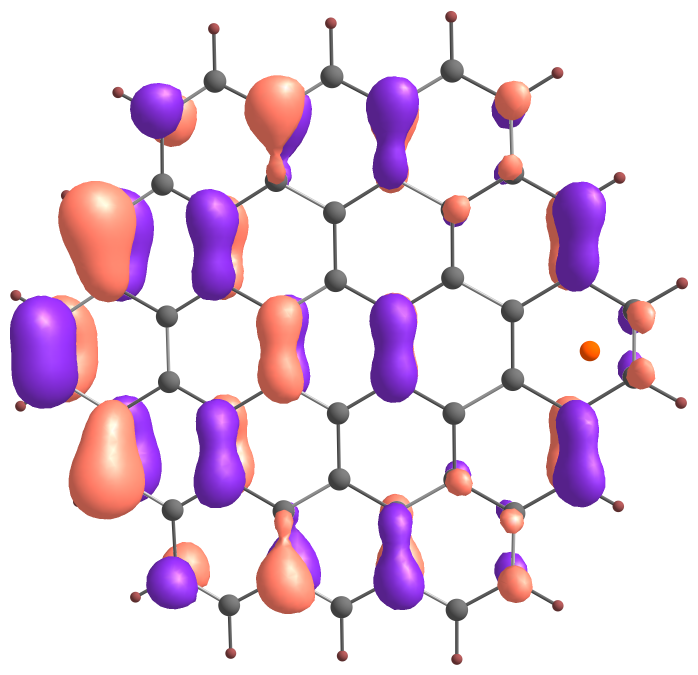 | 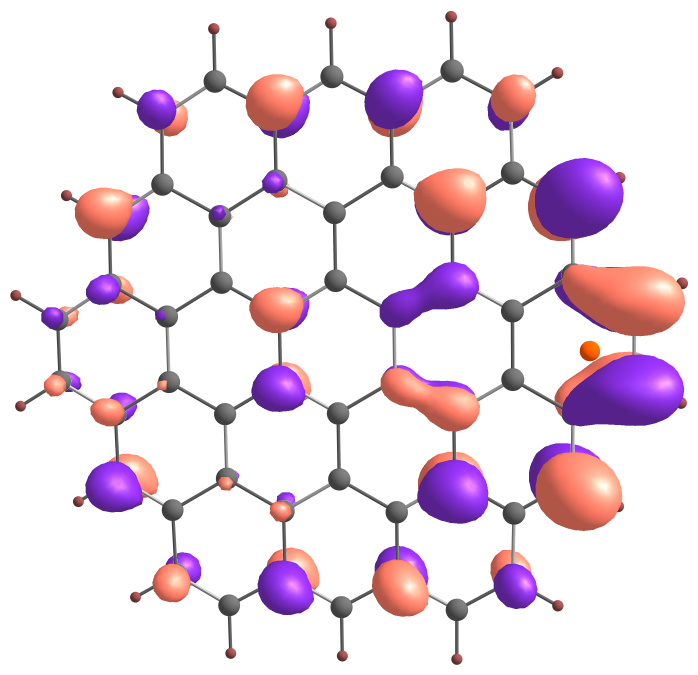 | 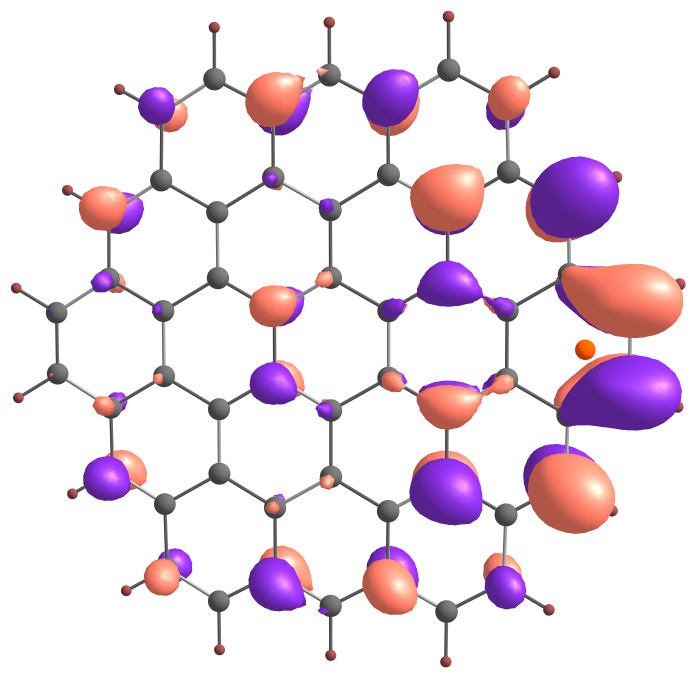 | 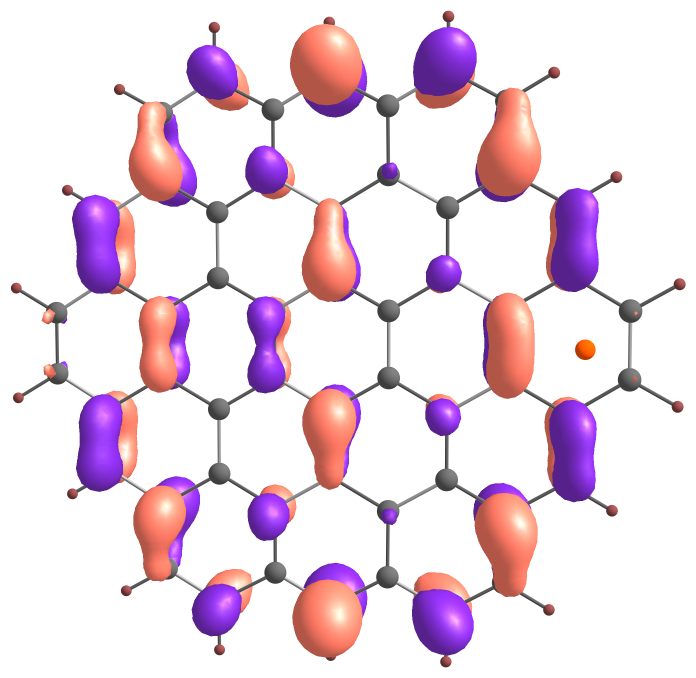 |
| C_54_H_18_  HOMO | C_54_H_18_  LUMO | Li^+^/C_54_H_18_  HOMO | Li^+^/C_54_H_18_  LUMO | Li/C_54_H_18_  HOMO | Li/C_54_H_18_  LUMO |
| 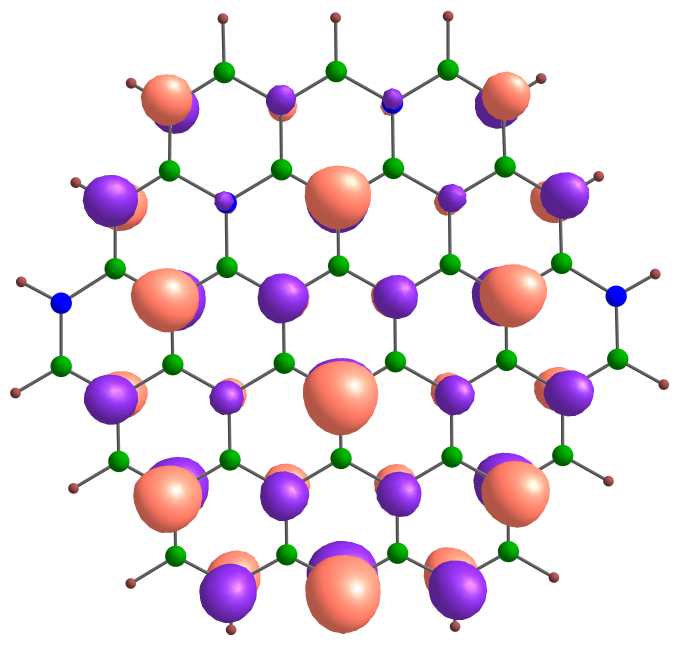 | 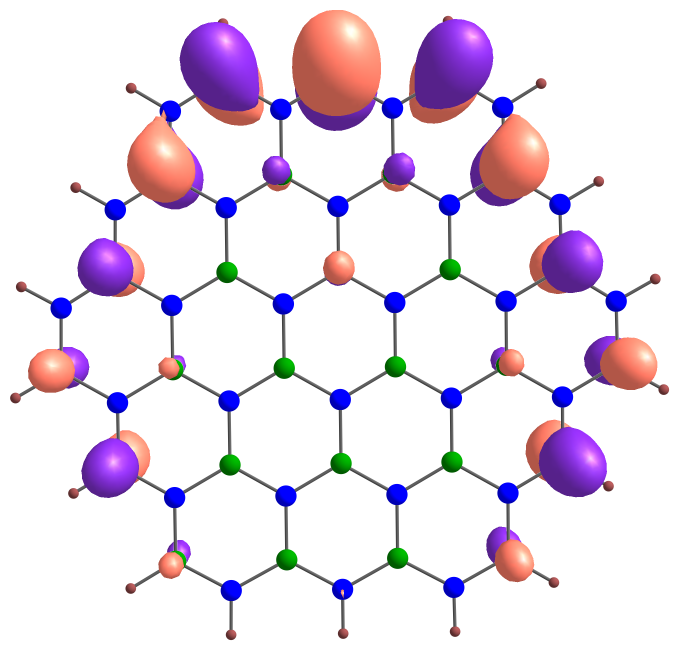 | 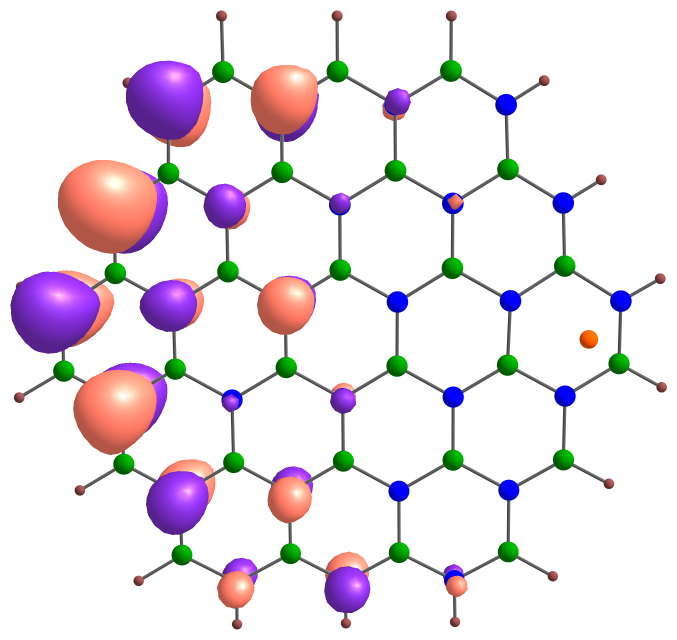 | 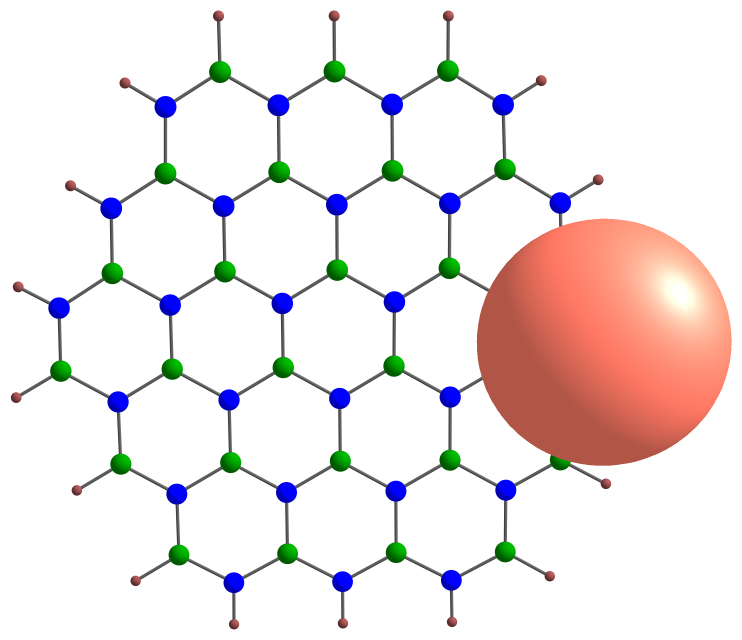 | 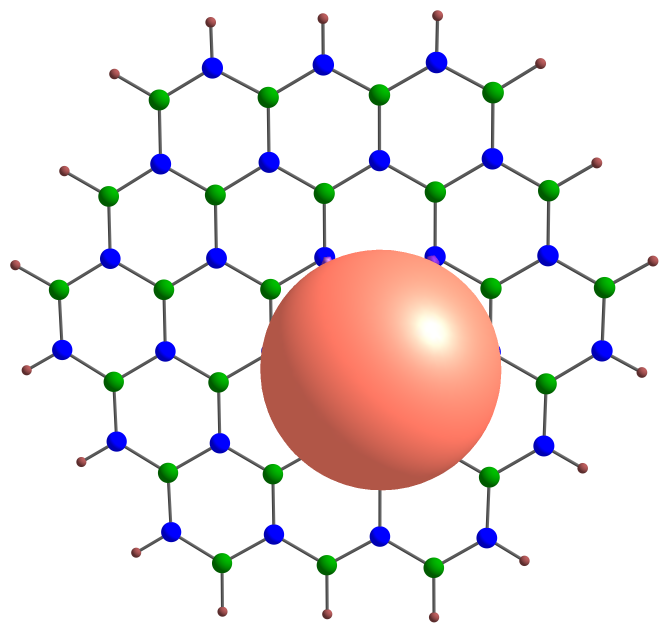 | 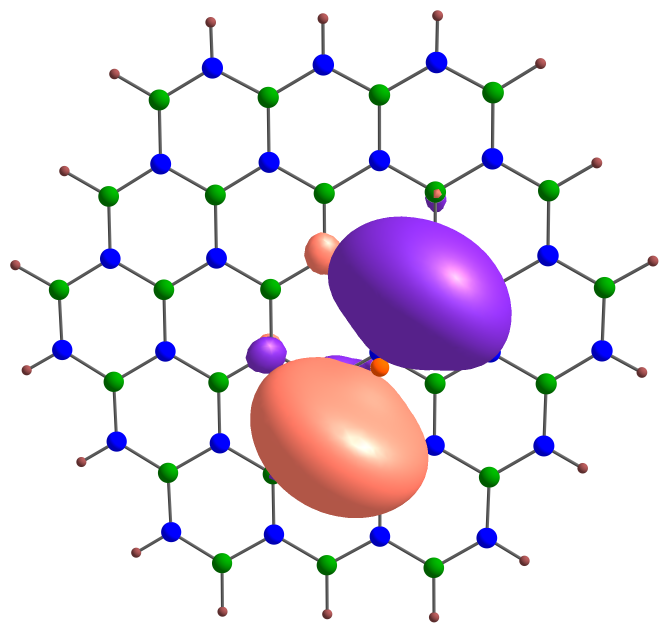 |
| B_27_H_18_N_27_  HOMO | B_27_H_18_N_27_  LUMO | Li^+^/B_27_H_18_N_27_  HOMO | Li^+^/B_27_H_18_N_27_  LUMO | Li/B_27_H_18_N_27_  HOMO | Li/B_27_H_18_N_27_  LUMO |

Figure S1. Molecular orbital diagrams for the representative pristine nanoflakes and the corresponding Li^+^- and Li-adsorbed systems.

| 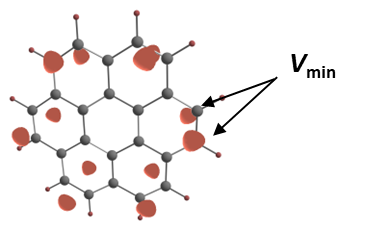 | 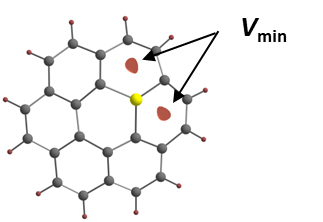 | 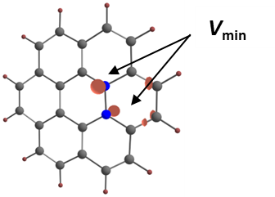 | 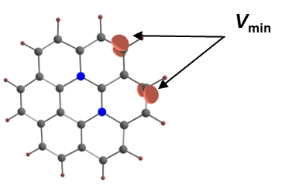 |
| --- | --- | --- | --- |
| (a) C_24_H_12_ | (b) C_23_H_12_Si | (c) o-C_22_H_12_N_2_ | (d) m-C_22_H_12_N_2_ |
| 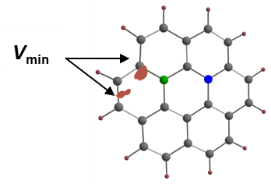 | 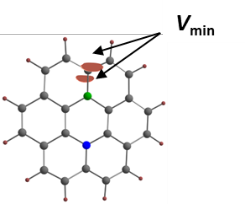 | 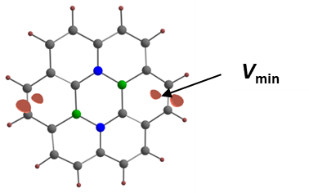 | 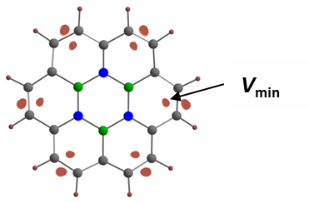 |
| (e) m-C_22_H_12_BN | (f) p-C_22_H_12_BN | (g) C_20_H_12_B_2_N_2_ | (h) C_18_H_12_B_3_N_3_ |
| 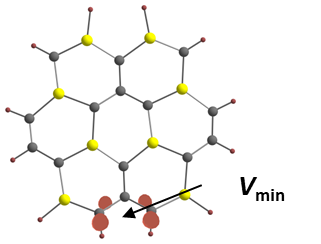 | 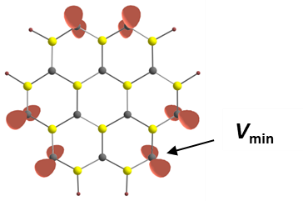 | 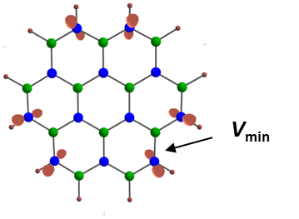 | 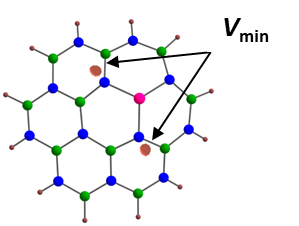 |
| (i) C_16_H_12_Si_8_ | (j) C_12_H_12_Si_12_ | (k) B_12_H_12_N_12_ | (l) B_11_H_12_N_12_Al |

Figure S2. MESP *V*_min_ features of (a) C_24_H_12_, (b) C_23_H_12_Si, (c) o-C_22_H_12_N_2_, (d) m-C_22_H_12_N_2_, (e) m-C_22_H_12_BN, (f) p-C_22_H_12_BN, (g) C_20_H_12_B_2_N_2_, (h) C_18_H_12_B_3_N_3_, (i) C_16_H_12_Si_8_, (j) C_12_H_12_Si_12_, (k) B_12_H_12_N_12_, (l) B_11_H_12_N_12_Al.


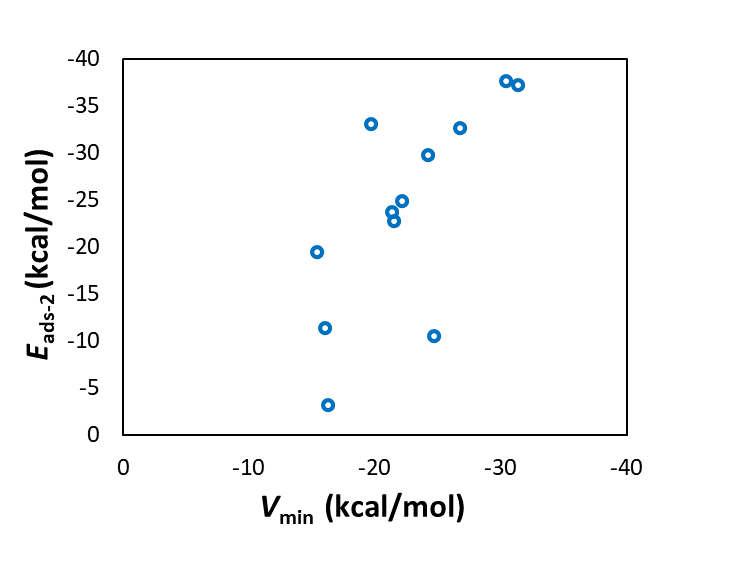

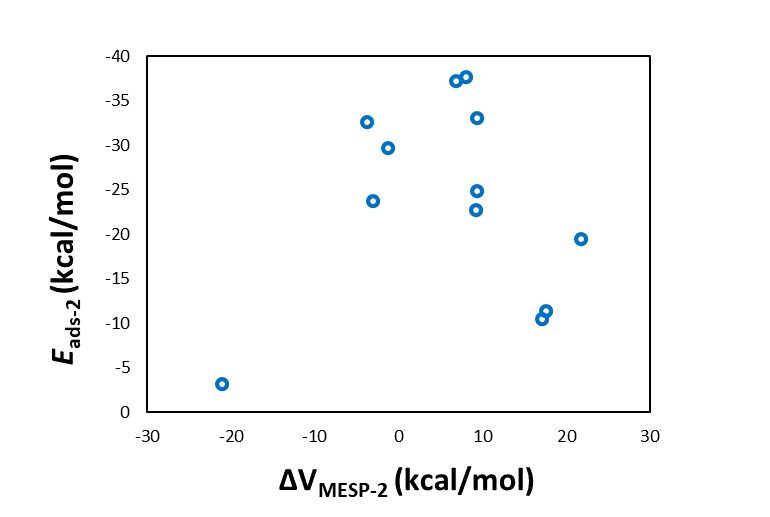


Figure S3. Correlation between *(a) V*_min_ and *E*_ads-2_ and (b) ∆*V*_MESP-2_ and *E*_ads-2_ for Li-adsorbed coronenes.


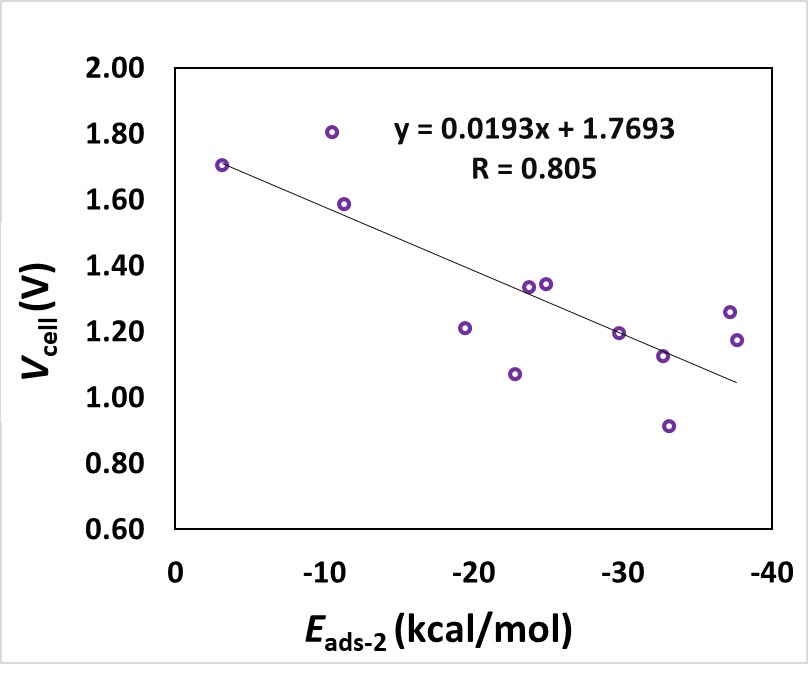
 **
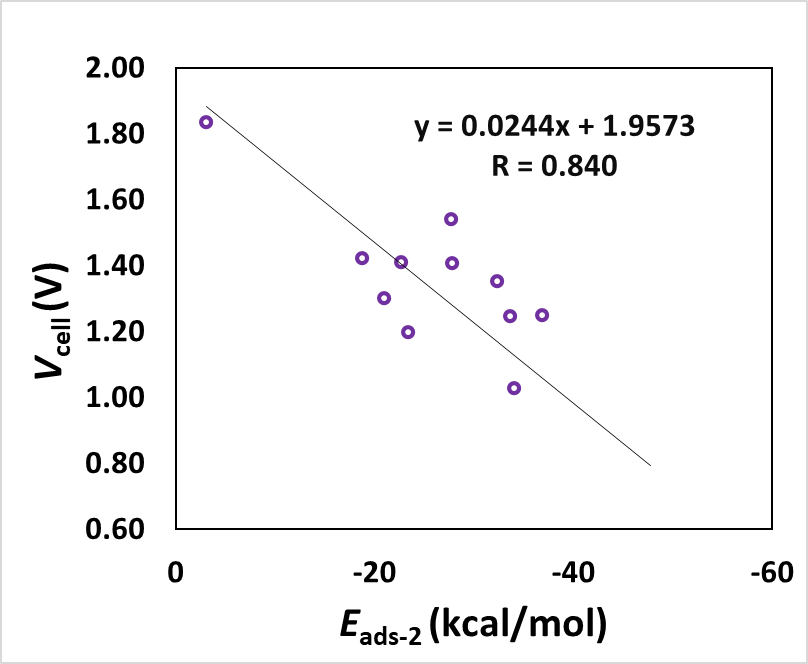
**

Figure S4. Correlation between  *E*_ads-2_ and V_cell_ for (a) doped coronene nanosheets and (b) doped circumcoronene nanosheets .

| 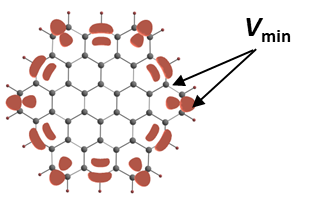 | 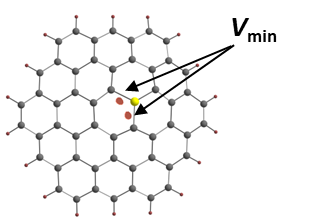 | 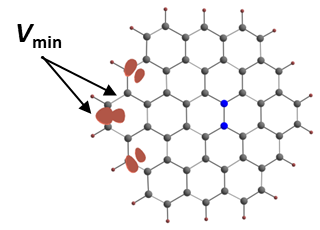 | 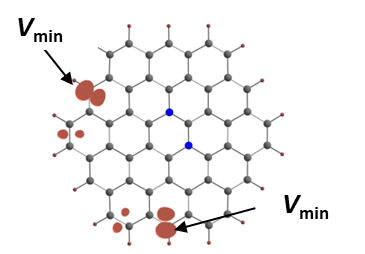 |
| --- | --- | --- | --- |
| (a) C_54_H_18_ | (b) C_53_H_18_Si | (c) o-C_52_H_18_N_2_ | (d) m-C_52_H_18_N_2_ |
| 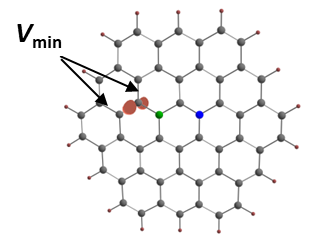 | 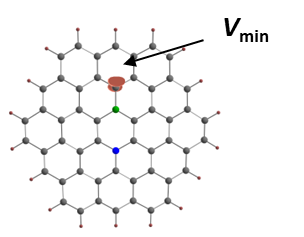 | 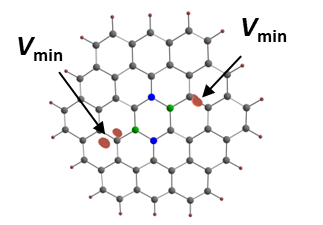 | 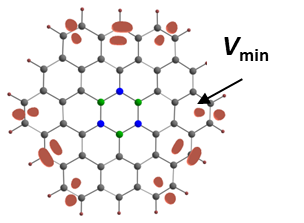 |
| (e) m-C_52_H_18_BN | (f) p-C_52_H_18_BN | (g) C_50_H_18_B_2_N_2_ | (h) C_48_H_18_B_3_N_3_ |
| 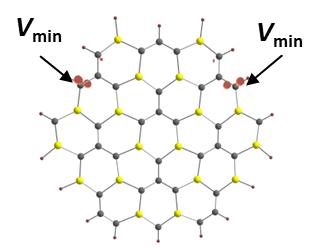 | 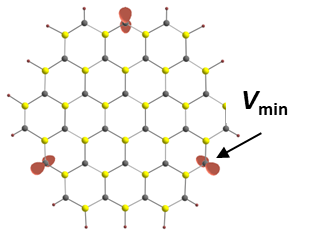 | 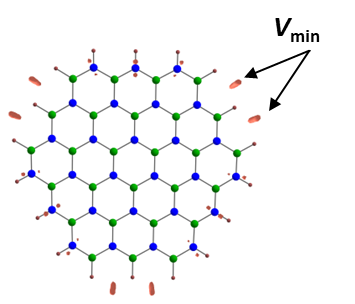 | 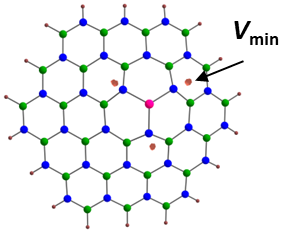 |
| (i) C_36_H_18_Si_18_ | (j) C_27_H_18_Si_27_ | (k) B_27_H_18_N_27_ | (l) B_26_H_18_N_27_Al |

Figure S5. MESP *V*_min_ features of a) C_54_H_18_, (b) C_53_H_18_Si, (c) o-C_52_H_18_N_2_, (d) m-C_52_H_18_N_2_, (e) m-C_52_H_18_BN, (f) p-C_52_H_18_BN, (g) C_50_H_18_B_2_N_2_, (h) C_48_H_18_B_3_N_3_, (i) C_36_H_18_Si_18_, (j) C_27_H_18_Si_27_, (k) B_27_H_18_N_27_, (l) B_26_H_18_N_27_Al.

**
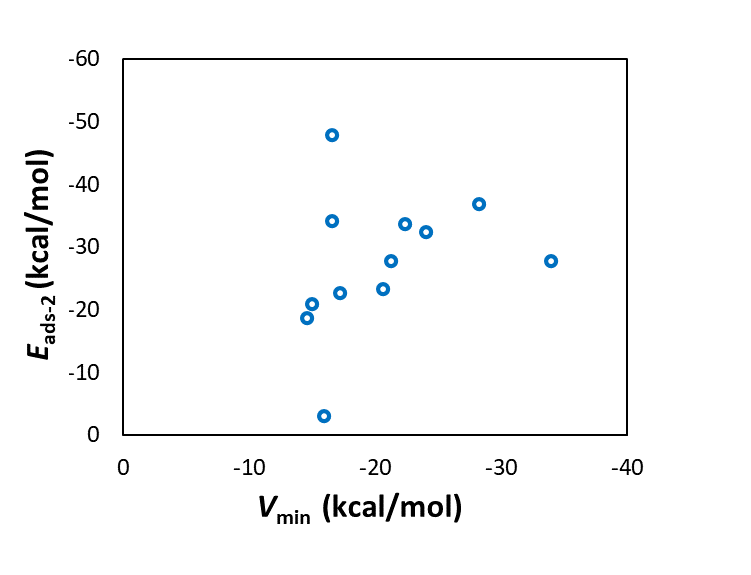

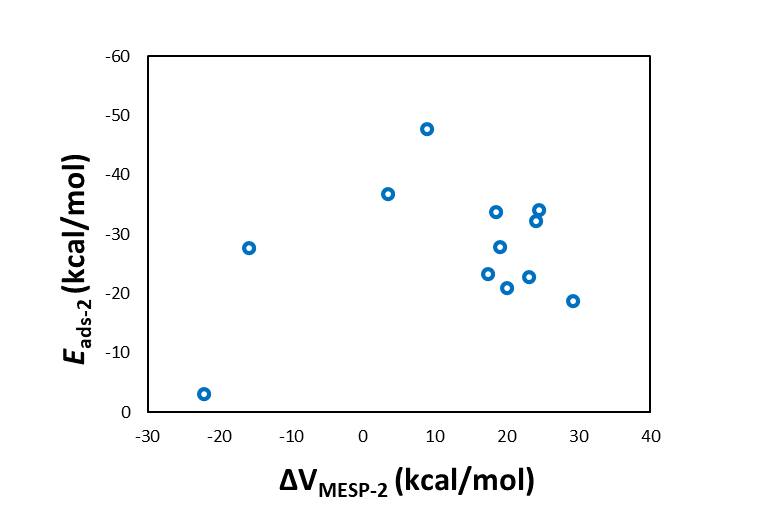
**

Figure S6. Correlation between *(a) V*_min_ and *E*_ads-2_ and (b) ∆*V*_MESP-2_ and *E*_ads-2_ for Li-adsorbed circumcoronenes.


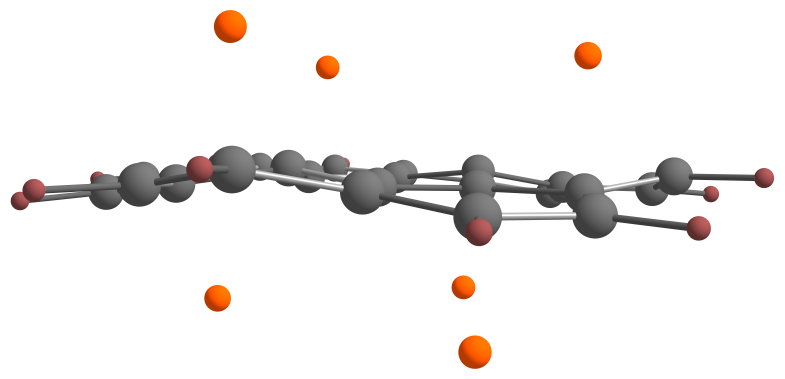


6Li@C_24_H_12_

Figure S7. Optimized structure of the 6Li@C_24_H_12_ system.

Table S1. The adsorption free energies (Δ*G*_ads_) for the adsorption of Li^+^ and Li on the nanoflakes. Values in kcal/mol.

|  | Li^+^ adsorption | Li adsorption |
| --- | --- | --- |
| nanoflake | ∆G_ads_ | ∆G_ads_ |
| C_24_H_12_ | -39.33 | -6.63 |
| C_23_H_12_Si | -44.97 | -25.34 |
| o-C_22_H_12_N_2_ | -47.37 | -21.97 |
| m-C_22_H_12_N_2_ | -44.00 | -16.16 |
| m-C_22_H_12_BN | -56.52 | -30.11 |
| p-C_22_H_12_BN | -55.03 | -30.66 |
| C_20_H_12_B_2_N_2_ | -46.58 | -18.07 |
| C_18_H_12_B_3_N_3_ | -38.58 | -13.38 |
| C_16_H_12_Si_8_ | -49.97 | -24.76 |
| C_12_H_12_Si_12_ | -43.29 | -3.69 |
| B_12_H_12_N_12_ | -33.69 | 3.29 |
| B_11_H_12_N_12_Al | -38.19 | -14.38 |
| C_54_H_18_ | -44.02 | -13.70 |
| C_53_H_18_Si | -50.25 | -40.49 |
| o-C_52_H_18_N_2_ | -46.35 | -14.23 |
| m-C_52_H_18_N_2_ | -53.46 | -24.11 |
| m-C_52_H_18_BN | -53.19 | -26.85 |
| p-C_52_H_18_BN | -51.06 | -21.20 |
| C_50_H_18_B_2_N_2_ | -48.80 | -26.74 |
| C_48_H_18_B_3_N_3_ | -42.42 | -14.37 |
| C_36_H_18_Si_18_ | -56.84 | -30.18 |
| C_27_H_18_Si_27_ | -53.83 | -19.80 |
| B_27_H_18_N_27_ | -36.40 | 3.71 |
| B_26_H_18_N_27_Al | -42.06 | -16.06 |
